# The Chromosome-level Genome Provides Insights into the Evolution and Adaptation of Extreme Aggression

**DOI:** 10.1093/molbev/msae195

**Published:** 2024-09-13

**Authors:** Peng-Cheng Liu, Zi-Yin Wang, Mei Qi, Hao-Yuan Hu

**Affiliations:** The School of Ecology and Environment, Anhui Normal University, Wuhu, Anhui Province, China; The School of Ecology and Environment, Anhui Normal University, Wuhu, Anhui Province, China; The School of Ecology and Environment, Anhui Normal University, Wuhu, Anhui Province, China; The School of Ecology and Environment, Anhui Normal University, Wuhu, Anhui Province, China

**Keywords:** parasitoid wasp, hi-C technology, energy metabolism, detoxification

## Abstract

Extremely aggressive behavior, as the special pattern, is rare in most species and characteristic as contestants severely injured or killed ending the combat. Current studies of extreme aggression are mainly from the perspectives of behavioral ecology and evolution, while lacked the aspects of molecular evolutionary biology. Here, a high-quality chromosome-level genome of the parasitoid *Anastatus disparis* was provided, in which the males exhibit extreme mate-competition aggression. The integrated multiomics analysis highlighted that neurotransmitter dopamine overexpression, energy metabolism (especially from lipid), and antibacterial activity are likely major aspects of evolutionary formation and adaptation for extreme aggression in *A. disparis*. Conclusively, our study provided new perspectives for molecular evolutionary studies of extreme aggression as well as a valuable genomic resource in Hymenoptera.

## Introduction

Aggressive behavior is important for animal survival, reproduction, and the acquisition of limited resources (Hardy and Briffa 2013). Aggression is a complex behavior and requires the integration of environmental and internal signals for effective behavioral output (Hardy and Briffa 2013, Yang et al. 2017). Many environmental factors (e.g. resource value, resource-holding potential), genes (e.g. *fruitless*, *Sry*), and neurotransmitters (e.g. serotonin, dopamine) control or influence the onset of aggression, aggressive behavior, and outcomes (Parker 1974, Enquist and Leimar 1987, Edwards et al. 2006, 2009, Dierick 2008, Siwicki and Kravitz 2009, Alekseyenko et al. 2013, Nijhout and Laub 2018). Remarkable advances in the study of aggression have been made in the fields of behavioral ecology, evolutionary biology, and molecular biology and in a wide range of species, from large mammals to small insects and even tiny nematodes (Hamilton 1967, Smith and Price 1973, West et al. 2001, 2002, Nelson 2005, Bailey et al. 2018).

Evolutionarily, selection should favor contestants that weigh the potential benefits and costs of conflict and adjust their behavior accordingly (Parker 1974, Arnott and Elwood 2009). As conflict is potentially damaging (even fatal) and energetically costly, individuals of most species (e.g. *Drosophila melanogaster*) usually avoid escalation and may give up and retreat before becoming injured. However, few species, mainly belonging to two groups of arthropods (Arachnoidea and Insecta), engage in unusually extreme aggression that results in severe injury or death of contestants (Enquist and Leimar 1990). Predictions from “hawk-dove” game theory suggest that extreme forms of aggression are an evolutionarily stable strategy only when critical resources are limited, and the benefits of winning far outweigh the potential costs of conflict (Smith and Price 1973, Enquist and Leimar 1987). Recently, studies of extreme aggression have mainly focused on behavioral evolution perspectives in a few species of aphids (Aoki and Makino 1982), spiders (Stoltz et al. 2008), Hymenoptera (Hamilton 1979, Murray 1987, Anderson et al. 2003, Innocent et al. 2011) and nematodes (Kapranas et al. 2020), while the molecular mechanism of this behavioral evolution is unknown.

Haplodiploid parasitoids are important insects that are well-known for their biological control of pests (Wang et al. 2019) and for theoretical research studies (Godfray 1994, Hardy and Wajnberg 2023). Competition for mating opportunities and resources (e.g. food, host) is common in parasitoids (Hardy and Briffa 2013). However, there are few reports and studies of species (e.g. *Melittobia australica*) that engage in extreme aggression (Enquist and Leimar 1990, Abe et al. 2003). *Anastatus disparis* (Ruschka) (Hymenoptera: Eupelmidae) is a generalist egg parasitoid of several Lepidopterans and is considered an important biological control agent for the severe forestry pests *Lymantria dispar* and *Dictyoploca japonica* in China and North America (Yan et al. 1989). Similar to most gregarious species (Godfray 1994), newly eclosed *A. disparis* males aggregate and await female eclosion near the emergence site, after which the females disperse after mating (i.e. local mate structure) (Hamilton 1967) due to the spatially aggregated hosts (e.g. egg masses of *L. dispar*). Adults can immediately complete mating after eclosion, and the progression of mating from receptive to post-mating lasts only several seconds (Liu et al. 2021). To acquire mating opportunities, this species frequently exhibits extreme aggression, such as severing their opponent's feet and antenna with their mouthparts, resulting in severe injury or even death after multiple attacks (Liu et al. 2017, Liu and Hao 2019, Fig. 1a). Aggression in *A. disparis* mainly included chasing (supplementary video S1, Supplementary Material online), sneak attacks by the opponent when courting a female (supplementary video S2, Supplementary Material online), and boxing (supplementary video S3, Supplementary Material online). Consistent with many empirical and theoretical reports (Smith and Price 1973, Enquist and Leimar 1990, Reinhold 2003, Innocent et al. 2011), local mate structure (resulting in mating opportunities limited in space or/and time) and short lifetime of male (∼ a week, resulting in low expected-lifetime mating success) are thought as vital factors contributing to the evolution of extreme aggression in *A. disparis* (Liu et al. 2017). Thus, *A. disparis* provides an excellent opportunity and experimental model to study the behavioral and molecular evolutionary biology of extreme aggression.

**Fig. 1.**
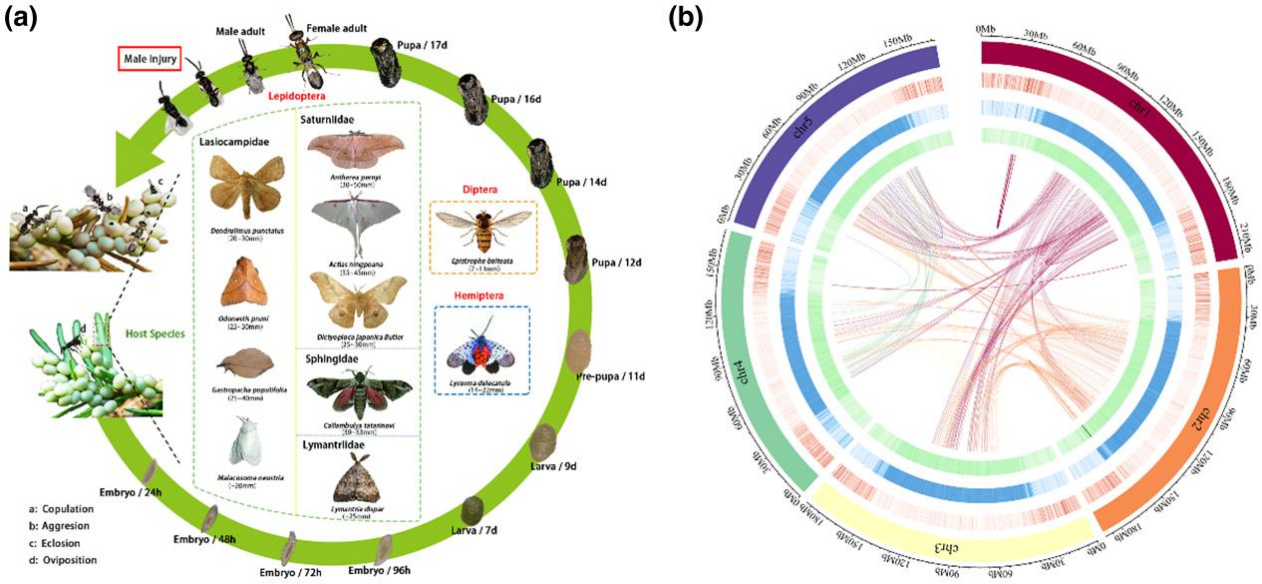
Life cycle and genome landscape of the egg parasitoid wasp *Anastatus disparis*. a) The developmental period from embryo to adulthood lasts approximately 18 d. Males experience earlier eclosion and remain near the emergence site to find mates and frequently suffer from extreme aggression that results in severe injury or even death. In addition, a) *disparis* has a wide range of host species, including 3 orders and 6 families. The body size of each host adult is provided below the Latin name. b) Tracks from outer to inner circles show five chromosomes at the Mb scale, the distribution of gene density, repeat density, GC density, and syntenic genes, with densities calculated in 0.1-Mb nonoverlapping windows.

Here, we report high-quality chromosome-level genomic resources of *A. disparis* that provide comprehensive insight into the molecular background of extreme aggression and the evolutionary basis of such adaptations. First, the basic genome description and comparative genomics of phylogenetic analysis, positively selected genes, and gene family expansion were investigated. Then, integrated with transcriptomics, we highlighted several important positively selected genes associated with one of the neurotransmitters dopamine synthesis, whose expression changed during the onset of aggression, and explored potential evolutionary mechanisms for extreme aggression in *A. disparis*. Besides, our results also suggested that energy metabolism especially from lipids and antibacterial activity, are possibly additional major aspects of extreme aggression adaptation. In summary, our study provides insights into the molecular and evolutionary mechanisms underlying the adaptation of *A. disparis* to extreme aggression and provides a valuable genomic resource for further research into the molecular basis of trait evolution in insects.

## Results and Discussion

### High-quality Genome of *Anastatus disparis*

Sequencing of the genome of the extremely aggressive parasitoid wasp *A. disparis* on the Nanopore platform generated 91.36 Gb of clean data with 97.23 × genome coverage after filtering out low-quality sequences and adaptor sequences (supplementary table S1, Supplementary Material online). After error correction of clean reads and assembly polishing, we obtained a high-contiguity genome assembly of 939.58 Mb consisting of 408 contigs with a contig N50 of 5.04 Mb (supplementary table S2, Supplementary Material online). The genome size of *A. disparis* is larger than most of the Hymenopteran genomes obtained to date (ranging from 180 Mb to 340 Mb [supplementary table S3, Supplementary Material online; Branstetter et al. 2018]). Consistent with the low guanine-cytosine (GC) content reported in hymenopterans (ranging from 30% to 45% [Standage et al. 2016]), the GC content of the *A. disparis* genome is 29.50%. GC-biased gene conversion and high recombination rates may be the reasons for the low GC content (Kent et al. 2012). To improve the genome assembly, Hi-C technology was used, and a total of 86 scaffolds representing 99.67% of the total assemblies were successfully anchored to 5 pseudochromosomes (Fig. 1b, supplementary table S4, Supplementary Material online). The length of the largest chromosome was 220.16 Mb, while that of the smallest was 170.35 Mb (Fig. 1b, supplementary table S5, Supplementary Material online). The scaffold N50 of the chromosome-level genome assembly reached 183.27 Mb. Based on both the *BUSCO* results (96.05% were found to be complete, 94.44% were single genes and 1.61% were duplicated genes) and mapping quality, the *A. disparis* genome assembly displayed high quality and completeness (supplementary table S6, Supplementary Material online); 99.38% of the de novo assembled transcripts were mapped to the reference genome (supplementary table S7, Supplementary Material online).

Extensive repeated sequences accounting for 65.23% of the whole genome (612.90 Mb, supplementary table S8, Supplementary Material online) were observed, especially transposable elements (TEs) with RNA transposons (408.10 Mb, 43.43% of the whole genome), which is higher than that in most sequenced Hymenopteran insects, e.g. *Nasonia vitripennis* (20.63%) and *Trichogramma pretiosum* (30.30%), and similar to that in *Gonatopus flavifemur* (60.70% [Yang et al. 2021]). Consistent with studies in many insects (Wang et al. 2014, Petersen et al. 2019, Wu and Lu 2019, Sun et al. 2021), the expansion of repeated sequences (mainly TEs with RNA transposons) may be one of the most important factors involved in increasing the size of the *A. disparis* genome. Besides, TE expansions and insertions can cause a variety of changes in the host genome and might contribute to adaptation, e.g. helping insects to increase insecticide resistance and adapt to climate change (González et al. 2010, Kim et al. 2014). The above environmental risks are frequently encountered by *A. disparis* due to the widespread application of insecticides and the wide distribution of its host species, from temperate to frigid zones (Sullivan et al. 1977, Yan et al. 1989).

In total, 19,246 protein-coding genes are predicted in the genome of *A. disparis* based on de novo prediction, homology alignment, and RNA-seq transcript assembly (supplementary table S9, Supplementary Material online, supplementary fig. S1, Supplementary Material online). We used *BUSCO* to evaluate the completion of our gene annotation and found that 96.20% (94% as single genes and 2.19% as duplicated genes) of the 1,367 genes were completely found (supplementary table S6, Supplementary Material online). The number of protein-coding genes in *A. disparis* was higher than that in most parasitoid wasps (supplementary table S3, Supplementary Material online), but lower than that in *N. vitripennis* (24,388 predicted genes). The predicted numbers of exons and introns are 104,333 and 85,087, respectively, with an average of 5.42 exons per gene and an average of 4.42 introns per gene (supplementary table S10, Supplementary Material online). Consistent with many eukaryotes, including Hymenopteran insects, the GC content varies greatly between different genomic regions (Louie et al. 2003, Rao et al. 2013, Kyriacou et al. 2024), and in our species, exons (36.8%) have a greater GC content than introns (25.1%). This phenomenon has also been observed in many studies and has been demonstrated to be significantly associated with various genomic features (e.g. gene density, recombination rate, distribution of repetitive elements, gene expression pattern [Eyre-Walker and Hurst 2001, Cohen et al. 2005, Rao et al. 2013]) and can be explained by several factors, e.g. mutational bias, base-specific excision repair of DNA mismatches, selection, and GC-biased gene conversion (Amit et al. 2012, Gelfman and Ast 2013, Figuet et al. 2014, Kyriacou et al. 2024). A total of 434 noncoding RNAs, including miRNAs (40), rRNAs (114), and tRNAs (280), were identified by in silico prediction (supplementary table S11, Supplementary Material online). In addition, 3,589 pseudogenes were predicted in the *A. disparis* genome, with an average length of 2621 bp (supplementary table S12, Supplementary Material online). Approximately 91.56% (17,621) of the predicted genes are functionally annotated in five public databases: nonredundant protein (Nr), Gene Ontology (GO), TrEMBL, euKaryotic Orthologous Groups (KOG), and KEGG Ortholog (KO [supplementary table S13, Supplementary Material online]). It will provide a comprehensive molecular basis for searching for possible evolution adaptations in *A. disparis*.

### Comparative Genomics

**Fig. 2.**
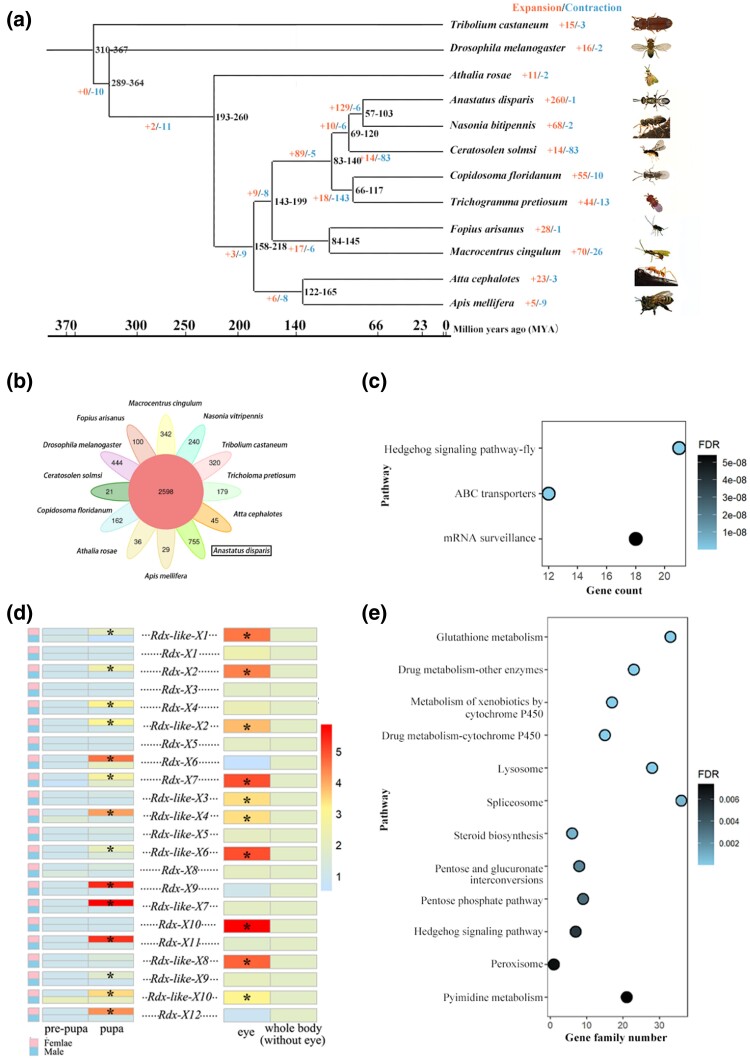
Comparative genomic analysis of the parasitoid wasp *Anastatus disparis* a) Maximum-likelihood tree showing the phylogenetic relationships of *A. disparis*, nine additional species of Hymenoptera, the fly *Drosophila melanogaster* and the beetle *Tribolium castaneum*. The phylogenetic tree was based on 1,760 single-copy proteins, with *T. castaneum* serving as the outgroup. The numbers of expanded (left) and contracted (right) gene families are shown on the branches. b) The petal figure informs about the number of gene families shared among the twelve investigated species, in which the central circle indicates the number of gene families shared by all species, and the edge circles indicate the number of species-specific gene families. c) Enriched KEGG pathways of a*) disparis*-specific genes. d) The expression patterns of *A. disparis*-specific genes annotated as roadkill genes (*Rdx*) that regulate the Hedgehog signaling pathway in females and males from the pre-pupal stage to the pupal stage and in the female eye at the pupal stage. Gene expression was determined through qRT‒PCR and calculated by the 2^−ΔΔCt^ method using the housekeeping gene *EF1A* as a reference. * indicates significantly different gene expression. e) Enriched KEGG pathways of expanded gene families in *Anastatus disparis.* The *KOBAS* software (v.2.0) was used to determine the significant enrichment of expanded genes in the KEGG pathways, and an adjusted *Q*-value < 0.05 was chosen as the significance cutoff.

To better understand the evolutionary events of the *A. disparis* genome, we performed a comparative analysis with 11 other insect (without extreme aggression) genomes containing 9 Hymenopterans (e.g. *Athalia rosae*, *Atta cephalotes*, *Apis mellifera*, *Macrocentrus cingulum*, *Ceratosolen solmsi*, *Trichogramma pretiosum*, *N. vitripennis*, *Copidosoma floridanum*, and *Fopius arisanus*), *D. melanogaster* and the red flour beetle *Tribolium castaneum*. The phylogenetic relationships among *A. disparis* and 11 other insect species (*T. castaneum*, as an outgroup) were determined according to a genome-wide set of 1,760 single-copy genes. Phylogenetic analysis by maximum likelihood (PAML) showed that five chalcidoids, *A. disparis*, *N. vitripennis*, *C. solmsi*, *T. pretiosum*, and *C. floridanum*, clustered together and that *A. disparis* had a closer relationship to *N. vitripennis* than to the other species. The estimated divergence times suggested that *A. disparis* diverged from the common ancestor of *A. disparis*-*N. vitripennis* ∼ 79 Mya (95% CI: 57 to 103; Fig. 2a).

We identified a total of 16,159 gene family clusters in our analysis (supplementary table S14, Supplementary Material online). A total of 2,598 gene families are shared by all selected 12 species and 755 *A. disparis*-specific gene families (i.e. lacking 1: 1ortholog in other species, containing 2,281 genes, supplementary File S1, Supplementary Material online) were identified (Fig. 2b). GO and KEGG enrichment analyses revealed that *A. disparis*-specific genes were enriched in eye formation (KO: 04341, Hedgehog signaling pathway-fly, *P* < 0.001; GO: 0042067, establishment of ommatidial planar polarity, *P* < 0.001 [Fig. 2c, supplementary fig. S2, Supplementary Material online]). Many *A. disparis*-specific genes annotated as roadkill genes (*Rdx*, also called HIB; *n* = 22) regulate the Hedgehog signaling pathway, which participates in the development of numerous tissues and organs (McMahon et al. 2003). This regulation may be important during eye formation for the proper packaging of ommatidia into a hexagonal array in flies. In humans, a dramatic effect of reduced Hedgehog signaling in embryos is cyclopia (Hooper and Scott 2005). Combined with the results of qRT‒PCR analysis, we found that, compared to those in the pre-pupal stage, most *Rdx* genes (14 out of 22) in the Hedgehog signaling pathway were significantly upregulated in females at the pupal stage (i.e. eye development and formation in this stage, according to the life cycle in Fig. 1a), and many *Rdx* genes (10 out of 22) were enriched in the female eye at the pupal stage (all *P* < 0.05, Fig. 2d). Sexual dimorphism in some wasp species (e.g. *Melittobia* spp.) is pronounced in the eye; males are blind, whereas females have fully functioning eyes (Freeman and Ittyeipe 1982, Matthews et al. 2009). Combined with most roadkill genes displaying sex- (i.e. female-) and tissue-specific expression during the pupal developmental stage, our genomic data suggest that *A. disparis* might exhibit distinct eye development or formation characteristics in females.

A total of 1,760 single-copy genes were screened for signatures of positive selection on the terminal branch of *A. disparis* in the phylogeny, as shown in Fig. 2a. The branch-site model in PAML was used to define the significance (*P* < 0.05, false discovery rate (FDR)-adjusted) of each candidate gene. In total, 354 genes showed evidence of positive selection in the *A. disparis* genome (supplementary File S2, Supplementary Material online). We did not observe any significantly enriched GO terms or KEGG pathways for these positively selected genes (*P* > 0.05).

We used *CAFE* software (v.4.2) to study gene family expansion and contraction during the evolution of *A. disparis* and related species. By comparing twelve species, we found that the *A. disparis* genome displayed one contracted and 260 expanded gene families (containing 2,466 genes, supplementary File S3, Supplementary Material online) compared to the common ancestor of *A. disparis* and *N. vitripennis* (Fig. 2a), and *N. vitripennis* contained two contracted and 68 expanded gene families. Comparatively, *A. disparis* showed more gene family expansions than did our other selected species in the order Hymenoptera. Enrichment analysis revealed that the expanded genes were mainly associated with detoxification (e.g. drug metabolism-other enzymes (KO: 00983, *P* = 2.46E−11), metabolism of xenobiotics by cytochrome P450 (KO: 00980, *P* = 6.19E−10), and drug metabolism-cytochrome P450 (KO: 00982, *P* = 4.67E−08 [Fig. 2e]). *Cytochrome P450* genes displayed distinct expansion, and 140 *cytochrome P450* genes were identified in the *A. disparis* genome, which is greater than the number found in the closely related wasp *N. vitripennis*. Phylogenetic analysis further indicates that *P450* underwent large expansions in the *A. disparis* genome (supplementary fig. S3, Supplementary Material online). *Cytochrome P450* enzymes can metabolize a broad range of endogenous and exogenous compounds, thus contributing to many processes, including nutrition provision, growth, development, xenobiotic detoxification, and pesticide resistance (Scott and Wen 2001, Feyereisen 2006). As the diverse host range of *A. disparis* contains 3 orders and 6 families (Fig. 1a), the expansion of *cytochrome P450* genes in *A. disparis* might have occurred during evolution to deal with different host metabolites (Ye et al. 2020). Along with *cytochrome P450* genes, the expansion of the detoxification family including *glutathione S transferase* (*GST*) (Salinas and Wong 1999, Song et al. 2020) and *ATP-binding cassette transporter* (ABC) (Roth et al. 2003, Wu et al. 2019), as well as the venom protein and chemosensory genes (data from supplementary File S3, Supplementary Material online) involved in host-parasite interactions may be explained by the species' diverse host range and complex living environment. In addition, this expansion might play an important role in the metabolic biotransformation of insecticides (Soderlund 1997). Thus, these potential genes might also be candidates for resistance in *A. disparis* that would improve its utilization for biological control and the evaluation thereof.

### Role of Dopamine in Aggressive Behavior

**Fig. 3.**
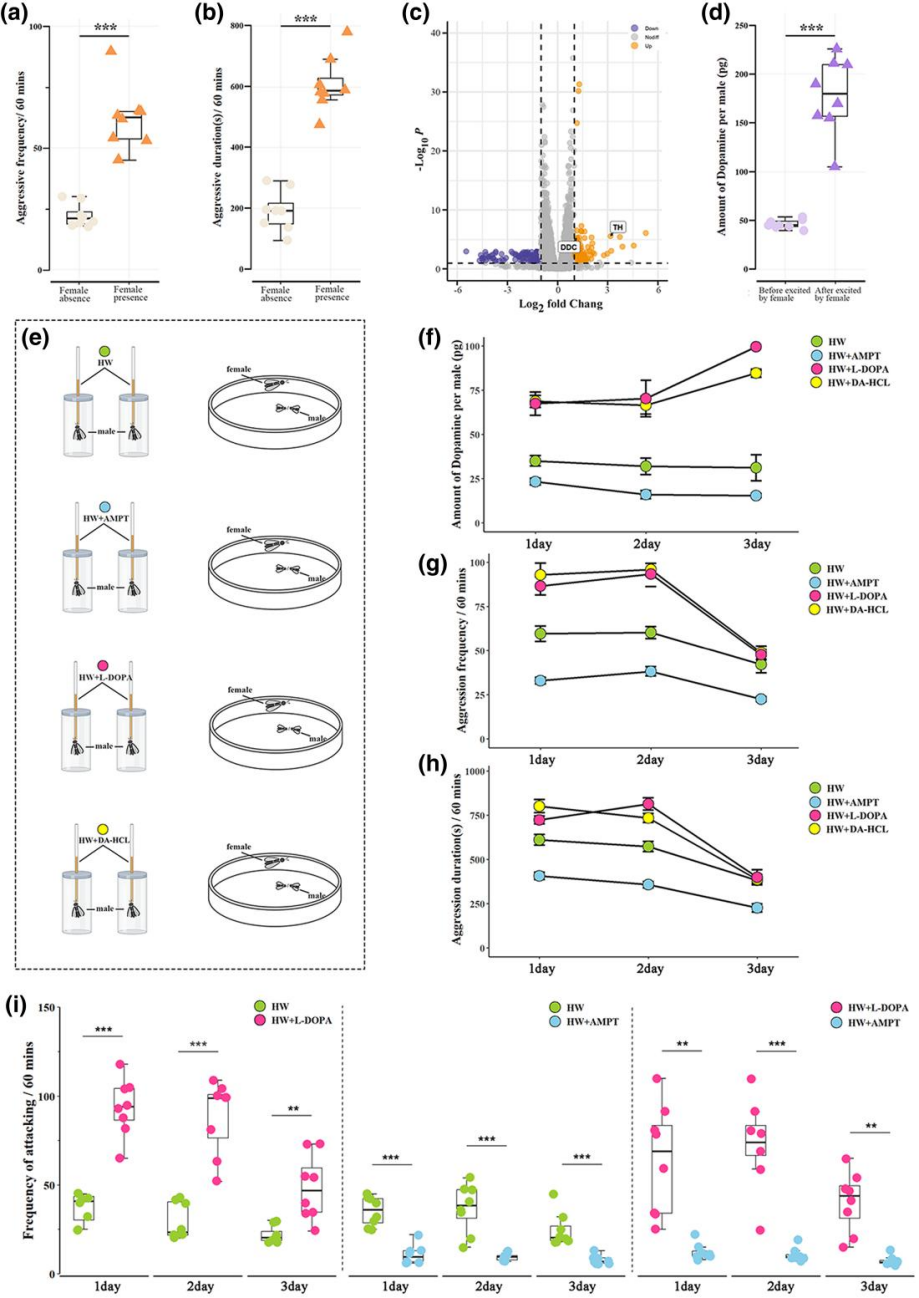
Role of dopamine in aggressive behavior. a) Boxplots describing the frequency of aggression for 60 mins under the conditions of female absence and female presence. Data point out of error bars is outlier. b) Boxplots describing the duration of aggression for 60 mins under the conditions of female absence and female presence. Data point out of error bars is outlier. c) Volcano plot showing differential gene expression between males excited by female for 30 mins and newly eclosed males. A total of 162 DEGs (differentially expressed genes) were found including 98 significantly upregulated genes (shown in yellow) and 64 significantly downregulated genes (shown in blue). d) Boxplots describing the dopamine levels in male brain excited by female for 30 mins. e) Charts for feeding males on honey water (HW), TH inhibitor AMPT, L-DOPA and DA-HCL, then aggressive experiments on males of the same treatment and age. f) Line charts showing dopamine levels in the male brain after feeding with HW, AMPT, or L-DOPA. g) Line charts showing the aggression frequency of two males of the same feeding state and age under the condition of female presence. h) Line charts describing the aggression duration of two males with the same feeding state and the same age under the conditions of female presence. i) Boxplots describing the attacking frequency of two males respectively feeding on different drugs (HW, AMPT, or L-DOPA) in an arena. *P < 0.05; **P < 0.01; ***P < 0.001.

Because access to females is important to males, being directly related to male fitness, male competition for mating opportunities is observed in almost all species (Takahashi et al. 2001, Riesch et al. 2006, Innocent et al. 2011). Expectedly, when mating opportunities were available in the environment (i.e. female was present in the arena), the frequency (Fig. 3a, *χ*^2^ = 4.563, *df* = 15, *P* < 0.001) and duration (Fig. 3b, *χ*^2^ = 7.206, *df* = 15, *P* < 0.001) of aggressive behaviors significantly increased approximately 3-folds, which a similar variation is also observed in the fighting intensity (Liu et al. 2017, Liu and Hao 2019). The transcriptomic analysis showed that 162 differentially expressed genes (DEGs) were found in males excited by females lasting for 30 mins, including 98 upregulated and 64 downregulated genes (Fig. 3c [SRA: PRJNA964572]). Only 5 DEGs experienced positive selection, and 2 out of 5 for biogenic amine synthesis, including the *TH* gene (Tyrosine hydroxylase, EVM0004425.1, also known as tyrosine 3-monooxygenase, encoding the dopamine synthesis rate-limiting enzyme, Budnik and White 1987) and *DDC* gene (EVM0013262.1, also known as aromatic L-amino acid decarboxylase, encoding the DOPA decarboxylase, which converts L-DOPA and L-5-hydroxytryptophan into dopamine and serotonin, respectively, as reviewed by Verlinden 2018 [data from supplementary File S2, Supplementary Material online]). In our study, the dopamine levels were significantly increased by ∼ 4-folds in males excited by females (Fig. 3d, *t* = 12.408, *df* = 13, *P* < 0.001), while there was no significant difference in serotonin (supplementary fig. S4, Supplementary Material online, *t* = 0.053, *df* = 8, *P* = 0.959). Dopamine is neuromodulatory and plays important roles in executive functions, motor control, motivation, arousal, reinforcement, and reward (Kume et al. 2005, Perry and Barron 2013, Costa and Schoenbaum 2022). Similar to serotonin, dopamine is known to modulate/control aggression in many species, e.g. *Drosophila*, ants (de Almeida et al. 2005, Alekseyenko et al. 2013, Kayser et al. 2015, Bubak et al. 2016, Asahina 2017, Yamaguchi and Lin 2018), which may result from the reduction in the sensitivity of the reward neurotransmitter system (Chen et al. 2005). Thus, we evaluated the role of dopamine in aggression in our species by feeding males on *TH* inhibitor α-methyl-p-tyrosine methyl ester (AMPT), L-DOPA, and Dopamine hydrochloride (DA-HCL) dissolving in 30% honey water (HW; Fig. 3e). Compared to those in the control group (i.e. feeding on HW), the amounts of dopamine in *A. disapris* males aged 1 to 3 d were, respectively, significantly downregulated after feeding on AMPT (*F*_(1, 43)_ = 20.706, *P* < 0.001) and upregulated after feeding on L-DOPA (*F*_(1, 43)_ = 45.753, *P* < 0.001) and DA-HCL (*F*_(1, 43)_ = 112.202, *P* < 0.001) (Fig. 3f). For the aggression experiment, two males of the same feeding state (i.e. AMPT + HW/L-DOPA + HW/HW + DA-HCL/HW) and of the same age were simultaneously placed into the arena with 1 female presence for 1 h. Expectedly, the aggressive frequency (Fig. 3g, *χ*^2^ = 78.604, *df* = 44, *P* < 0.001) and duration (Fig. 3h, *χ*^2^ = 56.437, *df* = 44, *P* < 0.001) of males feeding on AMPT were significantly decreased than that feeding on HW. While, the aggressive frequency and duration of males feeding on L-DOPA (*vs.* feeding on HW, frequency: *χ*^2^ = 30.063, *df* = 44, *P* < 0.001, duration: *χ*^2^ = 17.391, *df* = 44, *P* < 0.001) and DA-HCL (*vs.* feeding on HW, frequency: *χ*^2^ = 41.296, *df* = 44, *P* < 0.001, duration: *χ*^2^ = 18.788, *df* = 44, *P* < 0.001) were significantly increased, especially at age of 1 (L-DOPA: frequency, *χ*^2^ = 16.025, *df* = 14, *P* < 0.001, duration, *χ*^2^ = 7.802, *df* = 14, *P* = 0.005; DA-HCL: frequency *χ*^2^ = 18.485, *df* = 14, *P* < 0.001, duration, *χ*^2^ = 12.12, *df* = 14, *P* < 0.001) and two (L-DOPA: frequency, *χ*^2^ = 19.784, *df* = 14, *P* < 0.001, duration, *χ*^2^ = 17.654, *df* = 14, *P* < 0.001; DA-HCL: frequency *χ*^2^ = 50.985, *df* = 14, *P* < 0.001, duration, *χ*^2^ = 13.27, *df* = 14, *P* < 0.001), expect for the 3-day-old males (L-DOPA: frequency, *χ*^2^ = 0.962, *df* = 14, *P* = 0.327, duration, *χ*^2^ = 0.15, *df* = 14, *P* = 0.699; DA-HCL: frequency *χ*^2^ = 1.246, *df* = 14, *P* = 0.264, duration, *χ*^2^ = 0.019, *df* = 14, *P* = 0.89). However, we observed that the aggressive frequency and duration of 3-day-old males feeding on L-DOPA (frequency: *χ*^2^ = 73.642, *df* = 14, *P* < 0.001, duration: *χ*^2^ = 10.352, *df* = 14, *P* = 0.001) and DA-HCL (frequency: *χ*^2^ = 79.849, *df* = 14, *P* < 0.001, duration: *χ*^2^ = 13.915, *df* = 14, *P* < 0.001) were significantly increased than that feeding on AMPT. Consistent with the findings of a previous study (Liu et al. 2021), the aggressive frequency and duration significantly decreased with age in all four groups of males feeding on HW (frequency: *χ*^2^ = 32.726, *df* = 21, *P* < 0.001; duration: *χ*^2^ = 26.712, *df* = 21, *P* < 0.001); AMPT (frequency: *χ*^2^ = 34.208, *df* = 21, *P* < 0.001; duration: *χ*^2^ = 26.178, *df* = 21, *P* < 0.001); L-DOPA (frequency: *χ*^2^ = 138.82, *df* = 21, *P* < 0.001; duration: *χ*^2^ = 37.824, *df* = 21, *P* < 0.001); and DA-HCL (frequency: *χ*^2^ = 151.908, *df* = 21, *P* < 0.001; duration: *χ*^2^ = 43.416, *df* = 21, *P* < 0.001), which is likely caused by the energy available for aggressive behavior in aged males significantly decreasing. Besides, it might be the potential explanation that the aggressive frequency and duration of males feeding on L-DOPA and DA-HCL were not significantly different from those of the control group of 3-day-old males feeding on HW. We also found that the males feeding on L-DOPA displayed more aggressiveness launching more attacks than that feeding on AMPT, when an arena contained two males, respectively, feeding on HW and L-DOPA (day 1: *t* = 8.778, *df* = 14, *P* < 0.001, day 2: *t* = 7.166, *df* = 14, *P <* 0.001, day 3: *t* = 3.95, *df* = 14, *P* = 0.001), HW and AMPT (day 1: *t* = 7.015, *df* = 14, *P* < 0.001, day 2: *t* = 5.66, *df* = 14, *P <* 0.001, day 3: *t* = 4.899, *df* = 14, *P* < 0.001), and AMPT and L-DOPA (day 1: *t* = 3.582, *df* = 14, *P* = 0.003, day 2: *t* = 6.589, *df* = 14, *P* < 0.001, day 3: *t* = 4.014, *df* = 14, *P* = 0.001 [Fig. 3i]).

**Fig. 4.**
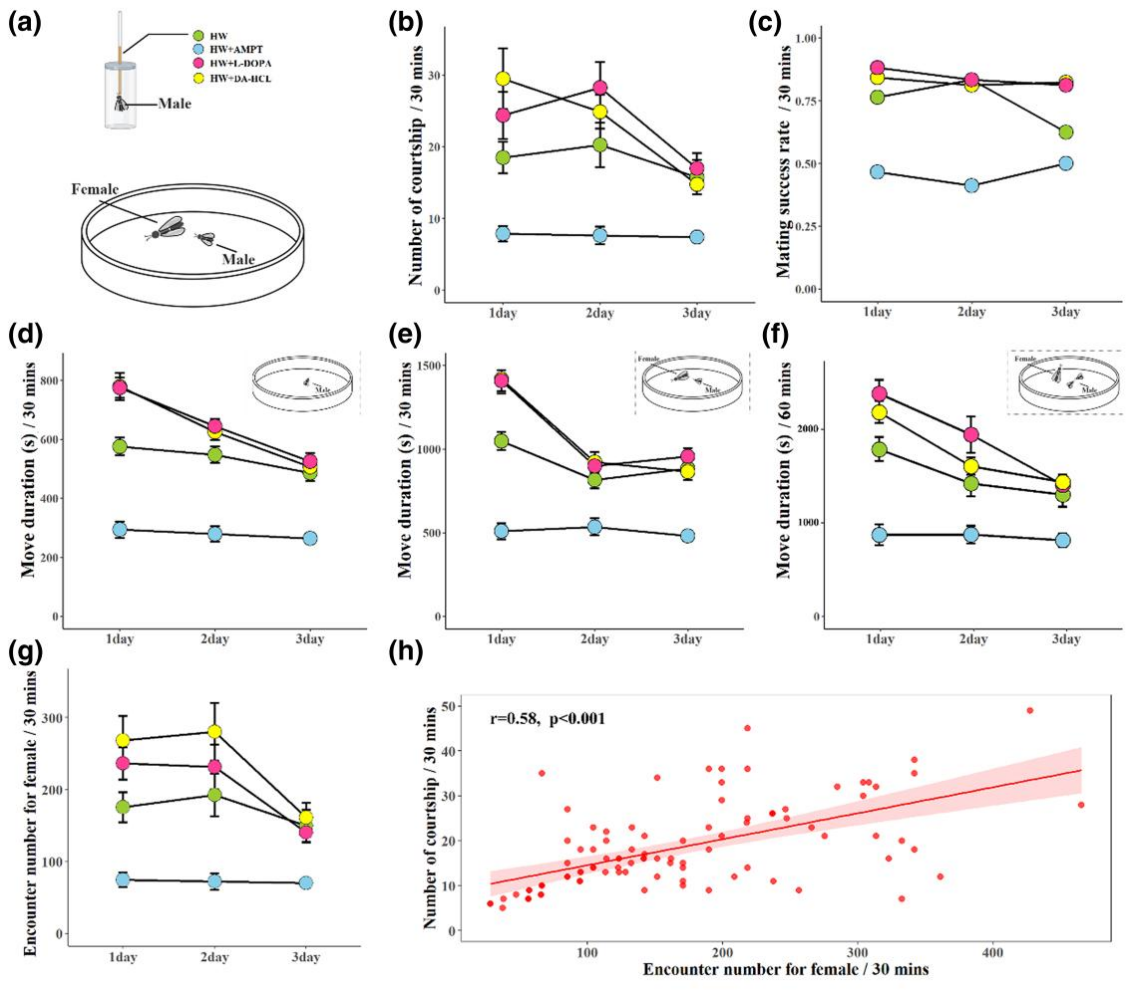
Role of dopamine in mating behavior and movement. a) Charts for male feeding and mating experiment. b) Line charts describing the courtship number of males during 30 mins after feeding on HW, AMPT, L-DOPA, and DA-HCL ranging from 1 to 3 d. c) The rate of successful mating of males during 30 mins after feeding on HW, AMPT, and L-DOPA. d) Line charts describing the move duration of the male during 30 min after feeding on HW, AMPT, L-DOPA and DA-HCL in the condition of the isolated male in an arena. e) Line charts describing the move duration of the male during 30 min after feeding on HW, AMPT, L-DOPA and DA-HCL in the condition of both female and male in an arena. f) Line charts describing the move duration of male during 60 mins after feeding on HW, AMPT, L-DOPA, and DA-HCL in the condition of one female and two males in an arena. g) Line charts describing the number of male (feeding on HW, AMPT, L-DOPA, and DA-HCL) encounters for female during 30 mins in the condition of both females and males in an arena. h) Pearson's correlation between the number of courtship and male encounter number for females during 30 mins.

**Fig. 5.**
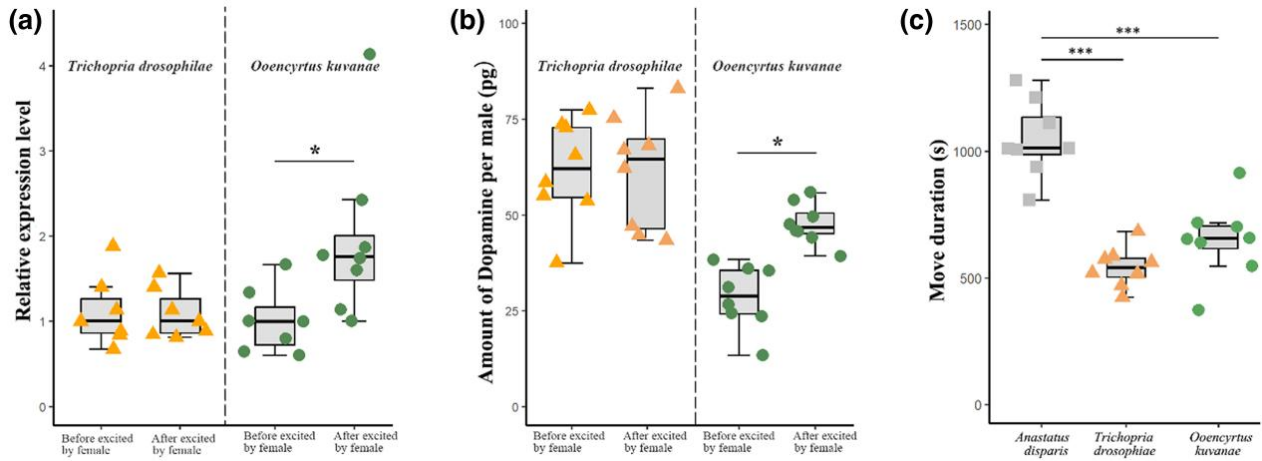
Dopamine levels in male brain and movement durations excited by female for 30 mins in other two parasitoids, *Trichopria drosophilae*, and *Ooencyrtus kuvanae.* a) Boxplots describing the expression of *TH* gene encoding the rate-limiting enzyme in dopamine synthesis were evaluated by qRT–PCR analysis. Data point out of error bars is outlier. b) Boxplots describing the dopamine were measured using an ELISA kit (BA E-5300, Germany). c) Comparison of the movement durations of *A. disparis*, *T. drosophilae*, and *O. kuvanae* males in the condition of both female and male in an arena. Data point out of error bars is outlier. *P < 0.05; **P < 0.01; ***P < 0.001.

Besides, dopamine is also shown to regulate male sexual behavior by modulating male arousal and visual perception during heterosexual courtship (Andretic et al. 2005, Kume et al. 2005), locomotor activity (Pendleton et al. 2002), and male courtship conditioning (Neckameyer 1998). Consistent with the effect of dopamine facilitating male sexual behavior (Melis and Argiolas 1995, Hull et al. 2004, Sasaki and Watanabe 2022), we also observed that, compared with male *A. disparis* feeding HW (Fig. 4a), male feeding L-DOPA and DA-HCL displayed more active courtship (Fig. 4b, L-DOPA, *c*^2^ = 5.027, *df* = 44, *P* = 0.025; DA-HCL, *χ*^2^ = 16.453, *df* = 44, *P* < 0.001) and a slightly greater rate of mating success (Fig. 4c, L-DOPA *vs*. HW at 1 d: 0.88 *vs*. 0.76, at 3 d: 0.81 *vs*. 0.63; DA-HCL *vs*. HW at 1 d: 0.84 *vs*. 0.76, at 3 d: 0.82 *vs*. 0.63, except for L-DOPA *vs*. HW at 2 d, 0.83 *vs*. 0.83 and DA-HCL *vs*. HW at 2 d, 0.81 *vs*. 0.83), while male feeding on AMPT displayed less active courtship (*χ*^2^ = 46.793, *df* = 44, *P* < 0.001) and significantly lower rate of mating success (AMPT *vs*. HW at 1 d, 0.47 *vs*. 0.76, at 2 d, 0.41 *vs*. 0.83, at 3 d, 0.5 *vs*. 0.63). In addition, similar to the regulation of locomotor activity by dopamine in honeybees and *Drosophila* (Pendleton et al. 2002, Akasaka et al. 2010, Riemensperger et al. 2011), *A. disparis* male fed L-DOPA and DA-HCL exhibited more active movement in the conditions of one female and one male simultaneously occurred in an arena (L-DOPA, *χ*^2^ = 13.054, *df* = 44, *P* < 0.001; DA-HCL, *c*^2^ = 8.117, *df* = 44, *P* = 0.004) and one female and two males in an arena (L-DOPA, *χ*^2^ = 12.325, *df* = 92, *P* < 0.001; DA-HCL, *c*^2^ = 6.351, *df* = 92, *P* = 0.012), even for isolated male (L-DOPA, *χ*^2^ = 17.779, *df* = 44, *P* < 0.001; DA-HCL, *c*^2^ = 11.865, *df* = 44, *P* = 0.001 [Fig. 4d to f]), and increased the frequency of encountering conspecific female (Fig. 4g, in the condition of one female and one male in an arena, DA-HCL, *c*^2^ = 7.416, *df* = 44, *P* = 0.006, except for L-DOPA, *χ*^2^ = 3.469, *df* = 44, *P* = 0.076). The opposite tendency was observed for male feeding on AMPT (move duration in three conditions, all *P* < 0.05; encounter frequency, *χ*^2^ = 33.123, *df* = 44, *P* < 0.001), and there was a moderate positive relationship between encounter frequency and courtship frequency (Fig. 4h, *r* = 0.58, *P* < 0.001). These results suggested that, similar to the findings of dopamine regulating locomotor activities for mating in male honeybees (Akasaka et al. 2010), dopamine affects *A. disparis* male mate behavior (i.e. more active courtship and greater proportion of mating success) is likely to credited to enhance male's movement and increasing frequency of encountering conspecific female. Accordingly, for acquiring valuable mating resources, overexpressed amounts of dopamine in contestants can not only increase the mating opportunity of acquisition by enhancing aggression but also significantly increase the individual's mating ability. For wider knowledge, other two parasitoids of *Trichopria drosophilae*, and *Ooencyrtus kuvanae* (without extremely aggressive behavior for mates) rearing in our lab were randomly selected to investigate the dopamine levels, and found that there was no significant difference (*T. drosophilae*, measured by the TH expression and dopamine amount, all *P* > 0.05) or only slightly increases (*O. kuvanae,* ∼1.6 folds, measured by the TH expression and dopamine amount, all *P* < 0.05) in males after excited by female (Fig. 5a and b). In addition, the movement durations of males after excitation by mating partner females in *T. drosophilae* and *O. kuvanae* were examined, and *A. disparis* males displayed more active movement (Fig. 5c, *A. disparis vs. T. drosophilae*, *t* = 8.453, *df* = 14, *P* < 0.001; *A. disparis vs. O. kuvanae*, *t* = 5.409, *df* = 14, *P* < 0.001). Our results suggest that dopamine overexpression in males excited by mating partner is likely to be the essential aspects for extreme mate-competition aggression formation and evolution in *A. disparis*. It might also provide a potential explanation that most species do not exhibit the extreme aggression pattern, although mates are vital and competition for mates is common in those species (e.g. randomly selected specie in our study). However, it is not yet clear whether abnormally overexpressed dopamine is the co-evolutionary pathway in other extreme mate-competition aggression species, more both non-extreme aggression and extreme aggression species need to be investigated for clarifying this puzzle. In any case, our study provided an example for the molecular evolution of extreme aggression behavior, especially the mate-competition.

Additionally, 27 *A. disparis*-specific genes were observed in the abovementioned 162 DEGs in males excited by females (data of specific genes from supplementary File S1, Supplementary Material online). However, the function of most specific genes is not well annotated, which limits our understanding of other aspects of the evolution and adaptation of extreme aggression. Some other genes were explored that have been proven to be associated with aggression. For example, two specific genes annotated neprilysin affecting the functioning of the nervous system were downregulated in excited males, which is consistent with the study of enhanced aggression in the mice's resident-intruder paradigm after neprilysin knockout (Siems et al. 2000). We also observed 5 DEGs annotated as *cytochrome P450* (3 out of 5 belong to CYP 4 family), that in *D. melanogaster*, *Cyp6a20* gene is proved to be directly involved in aggressive behavior (Dierick and Greenspan 2006). Besides, those genes that belonged to families also displayed obvious expansion (data from supplementary File S3, Supplementary Material online), and conclusively, all these provide valuable resources for us to further study the molecular mechanism and evolution of extreme aggression.

### Metabolic Genes Differentially Expressed During Male‒Male Aggression

**Fig. 6.**
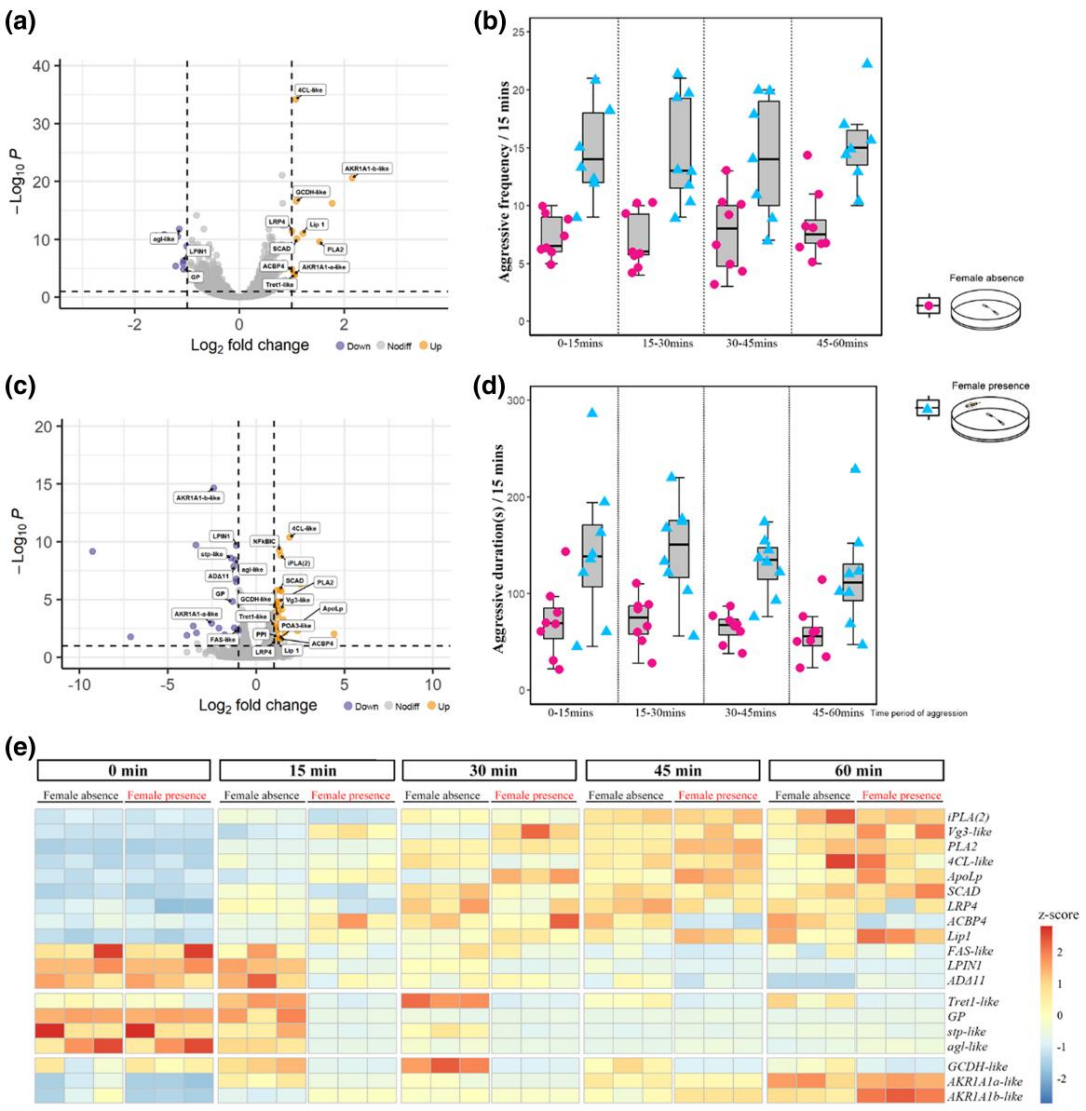
Behavioral aggression and gene expression patterns for metabolism under the conditions of female absence and presence. a) Volcano plot showing differential gene expression between males during aggression lasting for 30 mins and newly eclosed males. Compared to newly eclosed males without any aggressive experience, 20 DEGs (differentially expressed genes) were found in males during aggression lasting for 30 mins. There were 9 significantly upregulated genes (shown in yellow) and 11 significantly downregulated genes (shown in blue). b) Volcano plot showing differential gene expression between males during aggression lasting for 60 mins and newly eclosed males. Compared to newly eclosed males without any aggressive experience, 52 DEGs (differentially expressed genes) were found in males during aggression lasting for 60 mins. There were 22 significantly upregulated genes (shown in yellow) and 30 significantly downregulated genes (shown in blue). c) Boxplots describing the frequency of aggression on four consecutive timescales for 60 mins under the conditions of female absence and female presence. Data point out of error bars is outlier. d) Boxplots describing the duration of aggression on four consecutive timescales for 60 mins under the conditions of female absence and female presence. Data point out of error bars is outlier. e) The expression pattern of metabolic genes at 0, 15, 30, 45, and 60 min under the condition of female absence and presence. Gene expression was determined through qRT‒PCR and calculated by the 2^−ΔΔCt^ method using the housekeeping gene *EF1A* as a reference.

Typically, aggressive behavior is extremely energetically costly (Payne and Pagel 1997, Briffa and Elwood 2004, Ducrest et al. 2008, Biro and Stamps 2010, Reid et al. 2012, Grobler and Wood 2013). Thus, to investigate the metabolism genes involved in aggression, two males were placed into the arena for aggression lasting for both 30 and 60 mins and then collected for transcriptomic analyses. To be as sure as possible that differential gene expression is caused by aggression per se rather than other factors, newly eclosed males under the female absence condition were selected, and isolated males were treated as controls. Compared to the isolated males, transcriptomic analysis showed that 20 and 52 DEGs were found in males during aggression lasting for 30 mins and 60 mins, respectively (Fig. 6a and b) (SRA: PRJNA826118). GO enrichment analyses identified that enriched subcategories among DEGs are mainly involved in oxidoreductase activity and lipid transporter activity (supplementary table S15, Supplementary Material online). There were 19 genes encoding carbohydrate/lipid transporters and metabolic enzymes, and their expression was further verified at more time points of lasting aggression by qRT‒PCR analysis (Fig. 6). Specifically, several genes associated with glucose metabolism, such as *Tret1-like* (trehalose transporter 1-like, EVM0000934.1), *agl-like* (*α-glucosidase-like*, EVM0005860.1), *GP* (*glycogen phosphorylase*, EVM0014398.1), and *stp-like* (*sugar transporter-like protein*, EVM0011231.1), *GCDH-like* (*glucose dehydrogenase [acceptor]-like*, EVM0015375.1) were significantly upregulated during the first 30 min of aggressive behavior (i.e. early stage) but was then downregulated in the later aggression period. It suggested that carbohydrates (e.g. trehalose and glycogen) in *A. disparis* may be quickly consumed and provide indispensable energy for aggression, especially in early aggression (i.e. before 30 min in this study). In addition to carbohydrates, the number of genes associated with steatolysis and transportation, such as *ACBP4* (*acyl-CoA-binding domain-containing protein* 4, EVM0016316.1), *4CL4-like* (*4-coumarate: CoA ligase 1-like*, EVM0003417.1), *PLA2* (*phospholipase A2*, EVM0001048.1), *iPLA(2)* (*calcium-independent phospholipase A2*, EVM0004294.2), *Lip1* (Lipase1, EVM0011964.1), and *SCAD* (*short-chain acyl-CoA dehydrogenase*, EVM0010514.1), were consistently highly expressed from 15 mins to 60 mins, and the expression of the genes *ApoLp* (*Apolipophorins*, EVM0014364.1), *Vg3-like* (*vitellogenin-3-like*, EVM0007464.2), and *LRP4* (*low-density lipoprotein receptor-related protein* 4, EVM0015878.1) was upregulated in the later period of aggression. However, the expression of genes related to fatty acid synthesis, i.e. *FAS-like* (*fatty acid synthase-like*, EVM0009772.1), *ADΔ11* (*acyl-CoA-Δ(11) desaturase*, EVM0015082.1), and *LPIN1* (*phosphatidate phosphatase LPIN1*, EVM0015404.1), was consistently downregulated in aggressive males. In addition, we found that oxidative phosphorylation (the main pathway involved in energy metabolism)-related genes of *AKR1A1-like* (*alcohol dehydrogenase [NADP(+)]-like*, EVM0004274.3, EVM0017105.1) were upregulated throughout the period of aggression. These results suggested that, in addition to “carbohydrates,” the energy required to sustain and support aggression in *A. disparis* may also be provided by lipid metabolism. Besides, the expressions of the above metabolic genes were further expanded to the condition of more intense aggression, i.e. female presence in the male‒male fighting arena (Fig. 6c and d: frequency: *F*_(1, 63)_ = 8.205, *P* < 0.001; duration: *F*_(1, 63)_ = 36.831, *P* < 0.001). The above metabolic genes *PLA2*, *iPLA(2)*, *4CL-like*, *LPIN1*, *LRP4*, *ApoLp*, *Vg3-like*, *Tret1-like*, *FAS-like*, *AKR1A1-like*, *ACBP4*, and *GCDH-like* were also differentially expressed in aggressive males according to qRT‒PCR (Fig. 6e). Although genes related to lipid transport and metabolism were consistently highly expressed until the end of the experiment (i.e. 60 mins), the expression of sugar-related genes was downregulated earlier (i.e. after 15 mins) in the presence of females than in their absence. To support the significant increases in the intensity (Liu and Hao 2019) and frequency of male aggression in the presence of females (Fig. 6), carbohydrates (e.g. trehalose and glycogen) might be quickly consumed, and lipid metabolism might provide more energy during more intense and/or frequent aggression.

### Effect of *ApoLp* on Aggressive Behavior

**Fig. 7.**
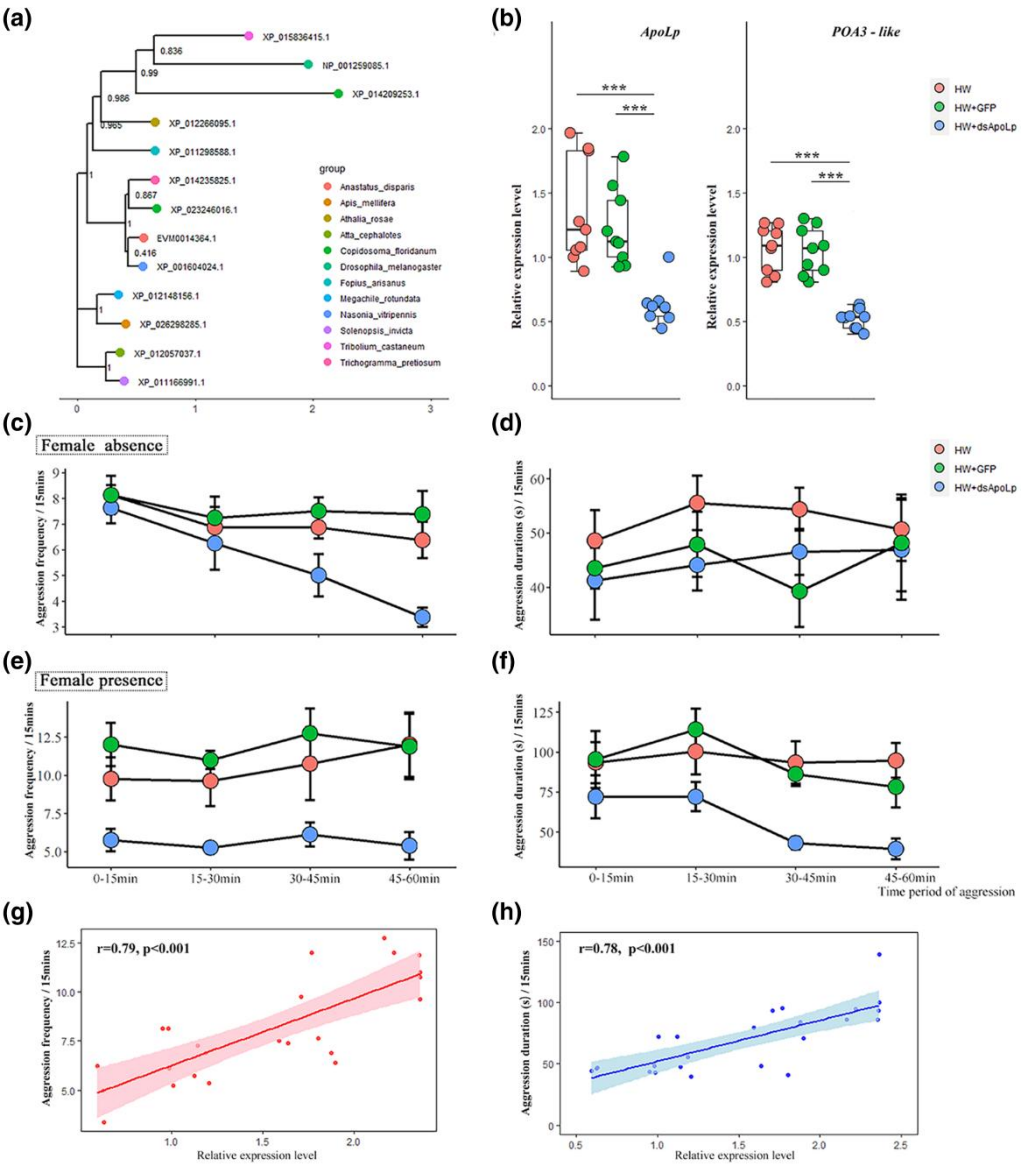
Decreased aggression after downregulation of *ApoLp* expression. a) The maximum-likelihood phylogenetic tree of *ApoLp* proteins in *Anastatus disparis* and eleven other insect species. The tree was constructed using IQ-TREE, and branch support was assessed by 1,000 ultrafast bootstrap replicates. The scale bar indicates the number of substitutions per site. b) The expression of *ApoLp* and *POA3-like* genes after males were fed HW (honey water), HW + *dsRNAGFP* (dsRNA of green fluorescent protein), and HW + ds*RNAApoLp*. Gene expression was determined through qRT‒PCR and calculated by the 2^−ΔΔCt^ method using the housekeeping gene *EF1A* as a reference. Data point out of error bars is outlier. c) Aggression was described according to the frequency of aggression under the condition of female absence. d) Aggression was described according to the duration of aggression under the condition of female absence. e) Aggression was described according to the frequency of aggression under the condition of female presence. f) Aggression was described according to the duration of aggression under the condition of female presence. g) Pearson's correlation between the frequency of aggression and gene expression of *ApoLp*. h) Pearson's correlation between aggression duration and gene expression of *ApoLp*. *P < 0.05; **P < 0.01; ***P < 0.001.

The above findings suggest that energy from the lipid metabolism process likely plays a role in sustaining and supporting intense/frequent extreme aggression in *A. disparis*. The *ApoLp* gene encoding apolipophorins is known to play an important role in lipid transport (Gupta et al. 2010, Zdybicka-Barabas and Cytryńska 2013). Thus, positively selected (data from supplementary File S2, Supplementary Material online) and male-biased *ApoLp* is an essential candidate gene related to aggression in *A. disparis*, and its role was tested as described below. Phylogenetic analysis showed that this gene is more closely related to that of *N. vitripennis* (Fig. 7a). After 3-day-old males were fed *dsRNAs* targeting *ApoLp*, the expression of the *ApoLp* gene was significantly downregulated by approximately 50% compared to that of males fed HW (*t* = 4.913, *df* = 16, *P* < 0.001) and HW + *dsRNA* of green fluorescent protein (*dsRNAGFP*) (*t* = 5.378, *df* = 16, *P* < 0.001) (Fig. 7b). The behavioral results showed that the frequency of aggression in males significantly decreased after downregulation of *ApoLp* expression in the condition of the absence of females (Fig. 7c, frequency: HW *vs*. HW + *dsRNAApoLp*: *χ*^2^ = 5.676, *df* = 56, *P* = 0.017; HW + *dsRNAGFP vs*. HW + *dsRNAApoLp*: *χ*^2^ = 9.676, *df* = 56, *P* = 0.002). Specifically, the frequency of aggression in males significantly decreased after 15 min of feeding on *dsRNAApoLp* (*vs*. after 45 min in the absence of females). While, the duration of aggression in males did not display significantly variation after downregulation of *ApoLp* expression in the absence of females (Fig. 7d, HW *vs*. HW + *dsRNAApoLp*: *χ*^2^ = 0.736, *df* = 56, *P* = 0.391; HW + *dsRNAGFP vs*. HW + *dsRNAApoLp*: *χ*^2^ = 0.148, *df* = 56, *P* = 0.7). Notably, the frequency and duration of aggression in males significantly decreased after the downregulation of *ApoLp* expression in the presence of females (Fig. 7e and f, frequency: HW *vs*. HW + *dsRNAApoLp*: *χ*^2^ = 46.145, *df* = 56, *P* < 0.001, HW + *dsRNAGFP vs*. HW + *dsRNAApoLp*: *χ*^2^ = 68.734, *df* = 56, *P* < 0.001; duration: HW *vs*. HW + *dsRNAApoLp*: *χ*^2^ = 10.939, *df* = 56, *P* = 0.001, HW + *dsRNAGFP vs*. HW + *dsRNAApoLp*: *χ*^2^ = 15.069, *df* = 56, *P* < 0.001). In addition, Pearson's correlation analysis revealed that the gene expression of *ApoLp* was moderately positively correlated with aggression frequency (Fig. 7g, *r* = 0.79, *P* < 0.001) and duration (Fig. 7h, *r* = 0.78, *P* < 0.001). The above results provide supporting evidence that genes related to lipid metabolism likely play a role in the aggressive behavior of *A. disparis*, especially for high-intensity aggression (i.e. in the condition of female presence).

**Fig. 8.**
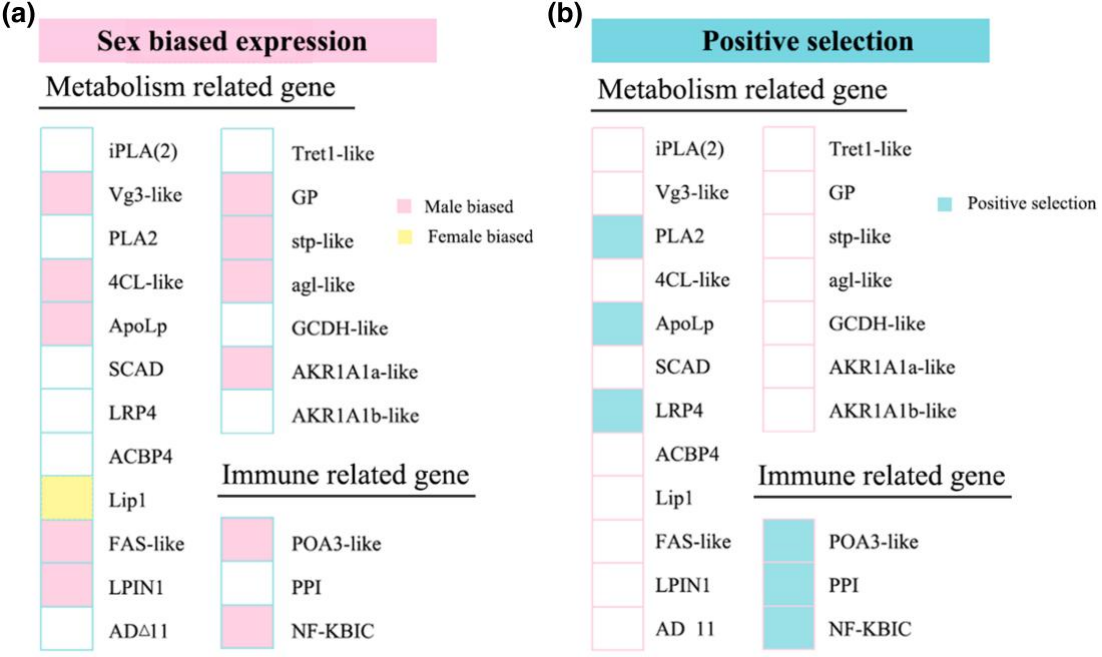
Sex-biased expression and positive selective genes related to aggression in the parasitoid wasp *Anastatus disparis* a) The expression pattern of metabolic and immune genes across different sexes indicated by transcriptomic data of female and male adults (SRA: PRJNA642922). b) Positively selected metabolic and immune genes detected on the *A. disparis* branch using *PAML* packages (v.4.9) with optimized branch-site mode (data from Supplementary File 2).

Energy is presumably the limited resource that motivates a large body of evolutionary theories focusing on energetic tradeoffs in behavior (Hoogenboom et al. 2013, Mathot and Dingemanse 2015). Aggressive behaviors are often positively correlated with a high metabolic rate (Payne and Pagel 1997, Briffa and Elwood 2004, Ducrest et al. 2008, Biro and Stamps 2010, Reid et al. 2012, Grobler and Wood 2013), and our study provides substantial evidence that numerous genes involved in carbohydrate/lipid oxidative metabolism are significantly differentially expressed during aggression in *A. disparis*. Typically, carbohydrate uptake and metabolism are important sources of energy that affect aggression in many species (Kemp 2002, Briffa and Sneddon 2007, Rittschof et al. 2015). In contrast, energy for sustaining and supporting extreme aggression in *A. disparis* is more likely provided by lipid oxidative metabolism (especially in males with decreased aggression after feeding on *dsRNAApoLp,* providing supporting evidence). Among the above 19 metabolic genes related to aggression, approximately half were male-biased genes (e.g. *vg3-like*, *4CL-like*, *ApoLp*, *FAS-like*, *LPIN1*, *AKR1A1-like*, *GP*, *agl-like*, and *stp-like* [Fig. 8a]; data from transcriptomic analysis, SRA: PRJNA642922), and 3 genes of *ApoLp*, *LRP4*, and *PLA2* associated with lipid metabolism were experienced positive selection (data from supplementary File S2, Supplementary Material online, Fig. 8b). Accordingly, genes involved in energy metabolism, especially from lipids, are possibly major aspect of extreme aggression adaptation for two reasons. First, similar to most gregarious species (Godfray 1994), *A. disparis* is a classic quasi-gregarious species in which females disperse after mating (Yan et al. 1989), and newly eclosed males aggregate and wait for female eclosion near the emergence site (i.e. local mate structure) (Hamilton 1967), during which male‒male chasing and aggression occur (Liu and Hao 2019). In this mate structure, males rarely leave the emergence site (Boulton et al. 2015), which leads to a lack of available exogenous carbohydrates, i.e. honeydew. Thus, evolutionary selection for the use of lipids as an important energy source for adaptation to extreme aggression in *A. disparis* might also be caused by the species' local mate structure. Second, the energy from oxidative metabolism is more than twice that produced by the oxidation of an equal weight of carbohydrates (Ranallo and Rhodes 1998). Due to the high intensity and high energy costs of extreme aggression (Fig. 3a and b, Fig. 6c and d), the consumption of more energy to sustain and support high-intensity extreme aggression might drive the force of evolution for the use of lipids as the main energy source in *A. disparis*.

### Upregulated Gene Related to Antibacterial Activity During Aggression

Besides, the upregulated genes in aggression lasting for 60 mins were also found to be involved in the immune response to bacterial genes, e.g. *NFkBIC* (*NF-kappa-B inhibitor cactus*, EVM0013578.1), *PPI* (*pacifastin-like protease inhibitor cvp4*, EVM0008147.1), and *POA3-like* (*phenoloxidase subunit A3-like*, EVM0007014.1) (Yu et al. 2018). In contrast to the aggressive behavior of most species, extreme aggression is usually characterized by conflicts that end with severely injured or dead contestants (Liu et al. 2017, Liu and Hao 2019). Consequently, injuries from extreme aggression might increase the probability of the individual being exposed to exogenous pathogens and activate the immune response in this case. Besides, *ApoLp* has been reported to increase antibacterial activity, participate in the activation of the prophenoloxidase cascade, and participate in cellular immune responses (Whitten et al. 2004, Yu et al. 2018). Consistent with these roles, the prophenoloxidase pathway might be activated through the interaction of *ApoLp* in *A. disparis* aggression, which prophenoloxidase gene, *POA3-like,* was significantly downregulated after males were fed *dsRNAApoLp* (HW *vs*. HW + *dsRNAApoLp*: *t* = 5.741, *df* = 10, *P* < 0.001; HW + *dsRNAGFP vs*. HW + *dsRNAApoLp*: *t* = 6.27, *df* = 10, *P* < 0.001) (Fig. 6b). Our result also showed that two genes of *NFkBIC* and *POA3-like* were biased expressed in males (data from transcriptomic analysis, SRA: PRJNA642922), and all genes (i.e. *POA3-like*, *PPI*, and *NF-KBIC*) were experienced positive selection (data from supplementary File S2, Supplementary Material online). It suggested that genes involved in antibacterial activity are possibly additionally specific aspects of extreme aggression adaptation. According to the transcriptomic analysis (SRA: PRJNA826557) in injured males for aggression (i.e. losers in a combat), genes of *NFkBIC*, *PPI*, and *POA3-like* were significantly upregulated, which provides substantial evidence.

## Conclusions

Currently, a large body of theoretical and empirical work in behavioral ecology has focused on the ways in which benefits/costs tradeoffs shape extreme aggressive behavior over evolutionary time (Smith and Price 1973, Enquist and Leimar 1987, 1990, Abe et al. 2005, Innocent et al. 2011, Stoltz et al. 2012, Mathot and Dingemanse 2015, Liu and Hao 2019, Kapranas et al. 2020), while the molecular mechanism of this behavioral evolution is unknown. Our study provides insight into molecular and evolutionary studies of extreme aggression in terms of neurotransmitter dopamine overexpression, energy metabolism, and energy sources. In addition, the frequency of injury from extreme aggression is also involved in the adaptation to aggression, as it activates antibacterial activity, which has rarely been studied in other aggression studies. Few insect species engage in extreme aggression (Hamilton 1979, Abe et al. 2003), and genomes of related species have not been provided. Therefore, a high-quality genome of *A. disparis* can provide a valuable genomic resource for further research on the molecular basis of extreme aggression trait evolution in insects, and other molecular bases of evolutionary adaptations such as venom function and behavioral host specificity.

## Materials and Methods

### Parasitoid

An *A. disparis* colony was first established from a population reared on *L. dispar* egg masses collected in March 2012 from Longhua County, Hebei Province, China (41°31′N, 117°74′E), and subsequently maintained on *A. pernyi* egg hosts. During the eclosion period, *A. disparis* adults from the 140th to 145th generations in the laboratory were collected for experiments. To minimize observer bias, blinded methods were used during material collection and subsequent measurements and analyses.

### DNA and RNA Extraction

Genomic DNA was extracted from approximately 100 female adults using the cetyltrimethylammonium bromide (CTAB) method. The quality and concentration of the extracted genomic DNA was assessed using 1% agarose gel electrophoresis and a Qubit fluorimeter (Invitrogen, Carlsbad, CA, USA). This high-quality DNA was used for subsequent Nanopore and Illumina sequencing. RNA was extracted with TRIzol Reagent (Invitrogen, USA), and first-strand cDNA was synthesized using a PrimeScript RT Reagent Kit (TaKaRa, Japan), which was used for subsequent transcriptomic analyses, dsRNA synthesis, and qRT–PCR.

### Genome Sequencing

A total of 15 µg of extracted genomic DNA was sheared using a Megaruptor (Diagenode, USA). The large DNA fragment was recycled by BluePippin (Sage Science, USA) and then subjected to DNA repair (NEBNext FFPE DNA Repair Mix) and dA tailing (NEBNext Ultra II End-Repair/dA-tailing Module). Then, ligation was performed by adding Adaptor Mix (SQK-LSK109, Oxford Nanopore Technologies), and a 1-µl aliquot was quantified with a Qubit 3.0 Fluorometer (Invitrogen, USA). The purified library was loaded onto flow cells for sequencing on a PromethION (Oxford Nanopore Technologies), generating raw data. Base-calling analysis of unprocessed data was conducted using Guppy software (v3.1.5).

### Genome Assembly and Scaffolding With Hi-C

Nanopore long reads, with a read N50 of 20,733 and a mean read length of 15,928 bp, were used for initial genome assembly. Error correction of clean data was conducted using *Canu* (v.1.5) (Koren et al. 2017), and then, the assembly was performed with *SMARTdenovo* software (https://github.com/ruanjue/smartdenovo). The consensus assembly was corrected with three rounds of *Racon* polishing (Vaser et al. 2017) and three rounds of *Pilon* polishing (Walker et al. 2014) using the Illumina reads with default settings.

The Hi-C technique was applied to improve the genome assembly at the chromosomal level (van Berkum et al. 2010). The Hi-C library was prepared using the standard protocol modified for application to whole insects (Xiao et al. 2020). In brief, 100 female adults were dissected into small pieces and immersed in 2% formaldehyde for crosslinking. Then, isolated nuclei were digested with the restriction enzyme Dpn II. The digested fragments were marked by incubation with biotin and ligated to each other to form chimeric circles. The biotinylated circles were sheared and sequenced using the Illumina HiSeq platform with 150-bp paired-end reads. In total, 623.65 million paired-end reads were generated from the libraries, and low-quality sequences and adaptor sequences were filtered out, generating clean data using *fastp* (Chen et al. 2018). By using *HICUP* (v.0.8.0), the clean paired-end reads were mapped to the draft assembled sequence (Wingett et al. 2015), and a total of 106.79 million uniquely mapped paired-end reads were generated, of which 74.41% were determined to be valid interaction pairs. Combined with the valid Hi-C data, *ALLHIC* (v.0.9.8) was applied to anchor the contigs onto the linkage groups using the agglomerative hierarchical clustering method (Zhang et al. 2019). A total of 532 contigs, representing 99.67% of the total assemblies, were successfully anchored to chromosome-level scaffolds (Burton et al. 2013). Finally, the scaffolds were concatenated with 100 Ns to create five pseudochromosomes.

BUSCO (v4.0) with 1,367 genes in Insecta OrthoDB (v10), was used to assess the completeness and accuracy of the assembled genome. To further assess its completeness, we employed Illumina RNA-seq data (SRA: PRJNA642922) to map the assembled genome using Bowtie2 software with default parameters (Langmead and Salzberg 2012).

### Repeat Annotation

Two complementary methods of de novo prediction and homology-based searches were used to predict repetitive sequences within the *A. disparis* genome. *De novo* repeat libraries were constructed using *RepeatScout* (v.1.0.5, Price et al. 2005) and *LTR-FINDER* (v.1.07, Xu and Wang 2007) with default parameters, and these repetitive sequences from the libraries were classified using *PASTEClassifier* (v.1.0, Hoede et al. 2014) with default parameters. Then, the de novo repeat libraries were combined with the Repbase database to obtain the final repeat database. Finally, *RepeatMasker* (v.4.0.5) with the parameters -nolow -no_is -norna -engine wublast was used to identify repeat sequences in the *A. disparis* genome by aligning them against the final repeat database (Tarailo-Graovac and Chen 2009).

### Protein-coding Genes, Noncoding RNA and Pseudogene Prediction

For the prediction of protein-coding genes in the *A. disparis* genome assembly, three methods of de novo prediction, homology-based search, and RNA-seq-based assembly were applied. Specifically, the *GENSCAN* (Burge and Karlin 1997), *Augustus* (v.2.4) (Stanke and Waack 2003), *GlimmerHMM* (v.3.0.4) (Majoros et al. 2004), *GeneID* (v.1.4) (Blanco et al. 2007), and *SNAP* (v.2006 to 2007-28) (Korf 2004) software programs were used for de novo prediction with the software's default parameters. For homology prediction, *GeMoMa* (v.1.3.1) (Keilwagen et al. 2016, 2018) software was used with amino acid sequences from *A. mellifera*, *A. rosae*, *N. vitripennis*, and *M. cingulum*. For the RNA-seq-based prediction, RNA-seq data from female and male *A. disparis* adults (three replicates for each sex, SRA: PRJNA642922) were mapped to the *A. disparis* genome using *HISAT2* (v.2.0.4) and assembled by *StringTie* (v.2.0), and *GeneMarkS-T* (v.5.1) was used to predict genes based on the assembled transcripts. In addition, gene prediction based on unigenes assembled by *Trinity* (v.2.11) was performed by *PASA* (v.2.0.2) (Campbell et al. 2006). Finally, all gene models were integrated using *EVM* (v.1.1.1) to obtain a consensus gene set (Haas et al. 2008). We evaluated the completeness of this final set of protein-coding genes with *BUSCO* (v.4.0), applying the protein mode by setting “-m proteins”.

MicroRNAs and rRNAs in the assembled *A. disparis* genome were identified by BLASTN searches against the Rfam database (Griffiths-Jones et al. 2005). tRNAs were predicted using tRNAscan-SE (Lowe and Eddy 1997). Candidate homologous gene sequences were identified by GenBlastA to mask protein-coding genes (She et al. 2009). Then, pseudogenes with a premature stop and/or frameshift mutation in the coding region were predicted by GeneWise (Birney et al. 2004).

Gene functions were inferred according to the best match of the alignments to the National Center for Biotechnology Information (NCBI) Nr, KOG, and TrEMBLdatabases using diamond blastp (diamond v2.0.4.142) and the KEGG database with an E-value threshold of 1E-5. GO IDs for each gene were obtained from TrEMBL. In total, approximately 17,621 of the predicted protein-coding genes could be functionally annotated with known genes, conserved domains, and GO terms.

### Comparative Genomic Analyses

Comparative genomic analysis was performed to analyze intraspecific gene duplication, explore the evolutionary relationships of different species, and identify species-specific genes. The analysis was based on *A. disparis*, and other 11 following genomes (species without extreme aggression): *A. rosae* (https://www.ncbi.nlm.nih.gov/datasets/genome/GCF_917208135.1/), *A. cephalotes* (https://www.ncbi.nlm.nih.gov/genome/? term=Atta+cephalotes), *A. mellifera* (https://www.ncbi.nlm.nih.gov/genome/48?genome_assembly_id=403979),

- *M. cingulum* (https://www.ncbi.nlm.nih.gov/datasets/genome/GCA_002156465.1/),
- *C. solmsi* (https://www.ncbi.nlm.nih.gov/genome/? term=Ceratosolen+solmsi), *T. castaneum* (https://www.ncbi.nlm.nih.gov/genome/? term=txid7070[orgn]), *D. melanogaster* (https://www.ncbi.nlm.nih.gov/datasets/genome/GCF_000001215.4/), *T. pretiosum* (https://www.ncbi.nlm.nih.gov/datasets/genome/GCF_000599845.2/), *N. vitripennis* (https://www.ncbi.nlm.nih.gov/genome/? term=Nasonia+vitripennis), *C. floridanum* (https://www.ncbi.nlm.nih.gov/genome/? term=Copidosoma+floridanum), and *F. arisanus* (https://www.ncbi.nlm.nih.gov/genome/? term=Fopius+arisanus).

Clusters of orthologous genes were classified using *OrthoFinder* (v.2.4, Emms and Kelly 2019) (diamond, e = 0.001) and annotated in the *PANTHER* database (v.15, Mi et al. 2019). As a result, 16,159 gene families were constructed, and 1,760 genes were identified as single-copy orthologous genes for phylogenomic analysis. For phylogenomic analysis, the amino acid sequences of each single-copy gene family were independently aligned by *MAFFT* (v.7.205) with the parameter –localpair –maxiterate 1000, filtered by *Gblocks* (v.0.91b) with the parameter -b5 = h, and then concatenated into one supersequence. Then, a species-level phylogenetic tree was constructed by ML using *IQ-TREE* (v.1.6.11) (Nguyen et al. 2015) with the best model (LG + I + G) estimated by modelfinder to evaluate evolutionary relationships. The *MCMCTree* (v.1.1) program of the *PAML* package was used to resolve the divergence times among species (Puttick 2019). Two calibration divergence time points based on fossil records from *TimeTree* (http://www.timetree.org/) (*A. mellifera vs*. *A. cephalotes*: 127 to 192, *T. castaneum vs*. *A. cephalotes*: 308 to 366) were used for divergence time calibration in our phylogenetic tree construction.

Positive selection signals were detected on the *A. disparis* branch through estimate therateratio (ω) of non-synonymous (Ka) to synonymous (Ks) nucleotide substitutions using *PAML* packages (v.4.9) with optimized branch-site mode. A likelihood ratio test was conducted to compare the alternative model and the null model. We calculated the *P* values by the chi-square test with the FDR, and genes with a *P*-adjusted value <0.05 were identified as positively selected genes. Gene family expansion and contraction were studied with *CAFÉ* software (v.4.2). For each branch and node of the phylogenetic tree, an expanded and contracted gene family with both a family-wide *P*-value and a viterbi *P*-value ≤ 0.05 was defined as a significantly expanded and contracted gene family (Han et al. 2013). KEGG enrichment analysis and annotation were performed using *clusterProfile* (v.3.14.0). To identify expanded *Cytochrome P450* gene family, protein sequences of well-annotated insects retrieved from UniProt were used as queries to search against the predicted protein sequences from *A. disparis* and selected insects using *BLASTP* (v.2.8.1, E-value 1e-5). The putative cytochrome *P450* protein was further checked for the presence of their characteristic domain of PF00067 from *Pfam*.

The expression of *A. disparis*-specific genes annotated as *Rdx* genes in females and males from pre-pupal to pupal and female eyes at the pupal stage was evaluated by qRT–PCR analysis (ABI StepOne Plus; USA) using TB Green Premix Ex Taq II (Takara, Tokyo). First, samples of *A. disparis* females and males at the pre-pupal (i.e. 11 d after egg laying) and pupal (i.e. 14 d after egg laying) stages were acquired by dissecting the parasitized host, and sex at the immature stage was determined by determining the size at which the female was significantly larger (supplementary fig. S5, Supplementary Material online). For acquiring the female's eye at the pupa stage, it was dissected from the pupa in PBS, and other tissues (e.g. head, thorax, and abdomen) were not further dissected because the whole body was merged, making it difficult to isolate. Each sample contained 20 individuals (*n* = 4), and RNA from each sample was extracted with TRIzol Reagent (Invitrogen, USA). Then, qRT–PCR amplification was conducted using a reaction mixture with 2 μL of cDNA template, 10 μL of 2×TB Green Premix Ex Taq II (Tli RNaseH Plus), 0.4 μL of 50×ROX Reference Dye, and 0.8 μL of each primer (10 μmol/μL), brought to a final volume of 20 μL by adding water. The cycling parameters were 95 °C for 30 s followed by 40 cycles of 95 °C for 5 s and 60 °C for 34 s, ending with a melting curve analysis (65 °C to 95 °C in increments of 0.5 °C every 5 s) to check for nonspecific product amplification. Relative gene expression was calculated by the 2^−ΔΔCt^ method using the housekeeping gene *translation elongation factor 1-α* (*EF1A*) as a reference to eliminate sample-to-sample variations in the initial cDNA samples. Primers for the genes were designed using Primer Express 2.0 software (supplementary table S17, Supplementary Material online).

### Aggressive Behavior Test

Aggressions in two conditions (the absence and presence of females) were assessed in this study. In each arena (height: 1 cm, diameter: 5 cm), two males (and one newly eclosed female in the female presence condition) were simultaneously placed into the arena for 1 h. The entire process was recorded using a video camera. The number of times and duration the males engaged in aggressive behavior (e.g. sneak attack, boxing, and chasing) were recorded in each arena.

### Genes Differentially Expressed in Male Excited by Female

Previous study demonstrates both virgin and mated female presence significantly increased and provoked male aggression versus that in the absence of females (Liu and Hao 2019). To acquire samples of aggressive males excited by females for detecting genes differentially expressed, 1-d-old virgin male was introduced into a petri dish containing 1-d-old mated female for 30 min and then collected for transcriptomic analyses (SRA: PRJNA964572). In this scenario, mated females were provided to avoid the differential expression of genes caused by male mating because females mate only once in their lifetime. For subsequent transcriptomic analyses, RNA for each sample (containing 15 to 20 males) was extracted with TRIzol Reagent (Invitrogen, Carlsbad), and 3 μg of total RNA was converted into cDNA using the TruSeq Stranded mRNA LT Sample Prep Kit (Illumina, San Diego) to construct cDNA libraries. To obtain raw reads, cDNA libraries were sequenced on an Illumina HiSeq X Ten platform, and 150-bp paired-end reads were generated. Then, reads containing adapters, poly-N reads, and low-quality reads were removed from the raw data by *FASTX-Toolkit* (https://github.com/Debian/fastx-toolkit), yielding clean reads. All clean reads were mapped to the *A. disparis* genome using *HISAT2* (v.2.0.4). Details of the transcriptome statistics (e.g. numbers of replicates, clean reads, mapped ratio) are listed in supplementary table S16, Supplementary Material online. The fragments per kilobase of transcript per million fragments mapped of each gene were calculated for the genes expressed using *StringTie* (v.2.0) (Florea et al. 2013). Genes with at least 2-fold expression changes and *P* (FDR-adjusted) < 0.05 as found by the *R package DESeq2* (v.1.22.1) were considered DEGs. The *GOseq R* package and *KOBAS* software were used to determine the significant enrichment of DEGs in the GO subcategories and KEGG pathways, and an adjusted *Q*-value < 0.05 was chosen as the significance cutoff.

### Effect of Dopamine on Aggressive and Mating Behavior

*TH* encodes what is thought to be the rate-limiting enzyme in dopamine synthesis (Budnik and White 1987). Thus, to reduce the biosynthesis of dopamine, we fed males the *TH* inhibitor AMPT (0.5 mg/ml [Sigma Aldrich]) dissolved in 30% HW. To increase DA levels, we fed males 10 mg/ml L-DOPA (Sigma Aldrich) and 0.1% L-ascorbic acid (Sigma Aldrich) dissolved in 30% honey water; L-ascorbic acid is used as an antioxidant (Liu et al. 2008, Bang et al. 2011). In addition, males were fed 2 mg/ml DA-HCL (Sigma Aldrich) dissolved in 30% honey water to directly increase DA levels. Since most mating occurs near the emergence site within 3 d of male eclosion and subsequent aggression also significantly decreases, virgin males were fed the above drugs at ages ranging from 1 to 3 d, and males with the same treatment and age were subsequently divided into two groups to conduct quantitative identification and behavioral experiments. Dopamine levels in *A. disparis* male brains were measured using an ELISA kit (BA E-5300, Germany) following the manufacturer's protocols. Each group contained 25 male heads, which were removed and dissected freshly in PBS.

For the aggression experiment, two males of the same age and in the same feeding state (i.e. HW/AMPT + HW/L-DOPA + HW/DA-HCL + HW) were simultaneously placed into the arena with one female presence for 1 h. The entire process was recorded using a video camera, and the number of times and duration the males engaged in aggressive behavior, movement duration, and number of encounters for each male were recorded accordingly. Besides, two males, respectively, feeding different drugs (HW and L-DOPA + HW, HW and AMPT + HW, AMPT + HW and L-DOPA + HW) were placed into the arena with female presence for 1 h and recorded who was attacked and the frequency. For easy observation, one of the males was randomly marked with white (with the other female marked with green) acrylic paint on the back of the thorax (Yin et al. 2023). To eliminate the effect of marker color on mate choice outcomes, reversing color marking was conducted in this experiment.

For the mating experiment, 1 male feeding on HW/AMPT + HW/L-DOPA + HW/DA-HCL + HW was introduced into a petri dish containing 1-d-old virgin female for 30 min (according to the data in previous studies, Liu et al. 2021, Yin et al. 2023, most males finish mating within 30 min after the experiment began), and recorded using a video camera. In each dish, whether and when successful mating occurred, stationary duration, courtship number and encounter number for females were determined by watching the recorded videos.

Finally, other two parasitoids of *T. drosophilae*, and *O. kuvanae* rearing in our lab were randomly selected to determine the dopamine levels in male brains after being excited by the female for 30 min (consistent with the period in *A. disparis* and many two species of males finish mating during this period according to the author's observation) using an ELISA kit (BA E-5300, Germany). Also, the expression of the *TH* gene encoding the rate-limiting enzyme in dopamine synthesis was evaluated by qRT–PCR analysis (ABI StepOne Plus; USA) using TB Green Premix Ex Taq II (Takara, Tokyo). Relative gene expression was calculated by the 2^−ΔΔCt^ method using the housekeeping gene *EF1A* as a reference. Primers for the genes were designed using Primer Express 2.0 software (supplementary table S17, Supplementary Material online).

### Genes Differentially Expressed During Male‒Male Aggression

To acquire samples of aggressive males for detecting genes differentially expressed during aggression, two males were placed into the arena for aggression lasting for both 30 and 60 min and then collected for transcriptomic analyses. To be as sure as possible that differential gene expression is caused by aggression per se rather than other factors, newly eclosed males under the female absence condition were selected, and isolated males were treated as controls. The method of transcriptomic analyses was consistent with the abovementioned method (supplementary table S16, Supplementary Material online [SRA: PRJNA826118]). The expression of selected genes was further evaluated by qRT‒PCR analysis using TB Green Premix Ex Taq II (Takara, Tokyo), which also expanded to more time points of aggression lasting for 15, 30, 45, and 60 min and the condition of more intense aggression (i.e. female presence in the male‒male fighting arena). Primers for the genes were designed using Primer Express 2.0 software (supplementary table S17, Supplementary Material online).

### Effects of *ApoLp* on Aggression

*dsRNA* targeting genes of *ApoLp* and *GFP* (green fluorescent protein) were synthesized with L4440 vectors containing a pair of oppositely oriented T7 promoters in HT115 (DE3) cell (Primers listed in supplementary table S18, Supplementary Material online). The integrity and concentrations of the synthesized *dsRNA* were determined by 1.5% agarose/Tris–acetate–EDTA gel and Nanodrop (Thermo Scientific Nanodrop 2000, Waltham [supplementary fig. S6, Supplementary Material online]). The accuracy of the synthesized *dsRNAApoLp* was checked by sequencing (453 bp), which completely matched the genome data. RNA interference was performed by feeding newly emerged males 10 ng/μL *dsRNAApoLp* or 10 ng/μL *dsRNAGFP* dissolved in 30% HW for 2 d. Then, the 3-d-old males were divided into two groups to conduct behavioral experiments for evaluating aggression and qRT‒PCR for checking the *dsRNA* effectiveness. Besides, phylogenetic analysis of the *ApoLp* genes was performed using ML methods with the best model estimated by *modelfinder* in *IQ-TREE* (v.1.6.11). Statistical support for the phylogenetic tree was assessed by ultrafast bootstrap analysis using 1,000 replicates.

### Statistical Analysis

All analyses were performed with *R* software (v.2.14.1). The qRT‒PCR data for roadkill genes, the *TH* gene, the *ApoLp* gene, and the *POA3-like* gene and the amount of dopamine in males after excitation by females were analyzed with an independent sample *t* test. The attack frequencies of two males that fed on different drugs in an arena were compared by paired-sample *t*-tests. Data on aggression frequency and duration in two conditions of female absence and presence, and male feeding on dsRNA were analyzed with generalized linear models (GLMs) using the lme4 package. Generalized linear mixed models (GLMMs) were applied to analyze the effects of male age and feeding different drugs on dopamine amount, aggression frequency and duration, courtship number, movement duration, and encounter number for a conspecific. In those models, male age and feeding of different drugs were treated as fixed effects, and the arena/group ID was introduced as a random effect. The aggression frequency, courtship number, and encounter number data were analyzed using a Poisson distribution (quasi-Poisson distribution for overdispersion) and log link function. Duration and amount of data were analyzed with a normal error structure and log transformation to normalize the residuals. The positive/negative relationships (e.g. between aggression frequency/duration and the expression level of *ApoLp*, between courtship number and encounter number for females) were assessed with Pearson's correlation analysis.

## Supplementary Material

msae195_Supplementary_Data

## Data Availability

The raw genome sequencing data have been deposited in the NCBI Sequence Read Archive (SRA) database (accession no. PRJNA693567). The final genome assembly was submitted to the NCBI with accession no. SAMN17864700. All transcriptome data are available through NCBI SRA (accession no. PRJNA642922, PRJNA964572, PRJNA826118, PRJNA826557).
