# Supplementary material for: The Chromosome-level Genome Provides Insights into the Evolution and Adaptation of Extreme Aggression
Source: Mol Biol Evol. 2024 Sep 13;41(9):msae195. doi: 10.1093/molbev/msae195 (PMC11427683; doi:10.1093/molbev/msae195)
Supplement: msae195_Supplementary_Data [file msae195_supplementary_data.zip › Supplementary Figure.pdf]

Supplementary Figure

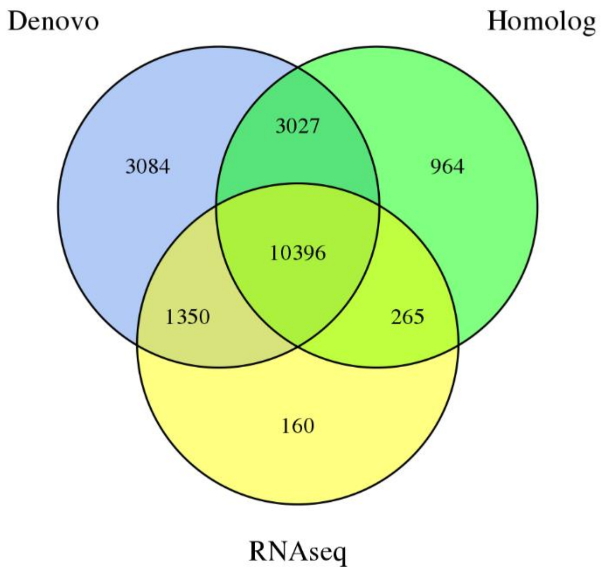

Figure S1. Venn diagram of predicted genes in *Anastatus disparis* genome. Three approaches of *de novo* prediction, homology alignment and RNA-seq transcript assembly were used to predicted genes in the genome of *A. disparis*.

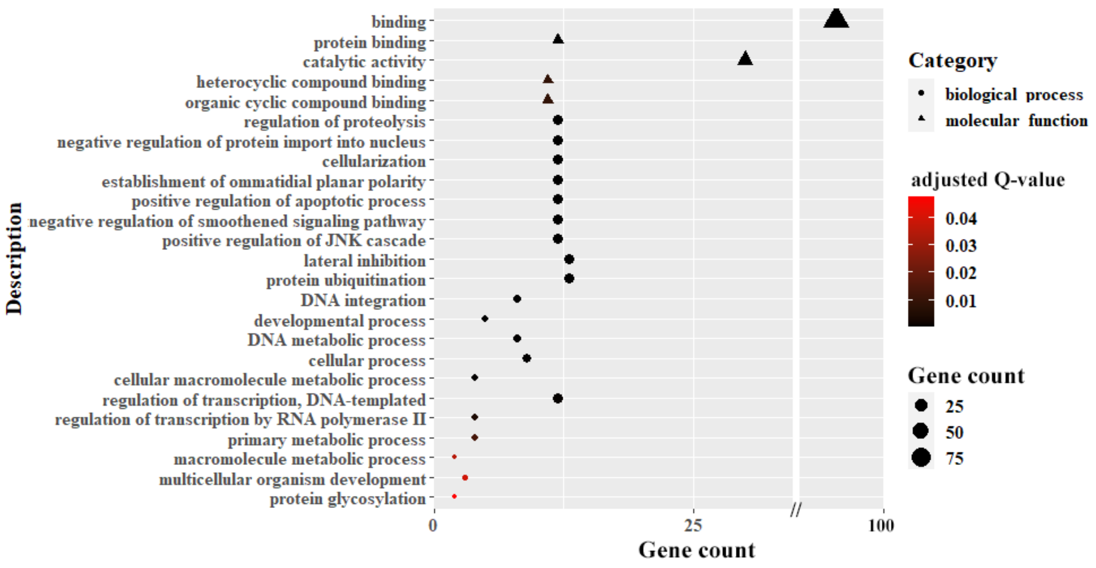

Figure S2. Enriched GO category of *Anastatus disparis*-specific genes. The *GOseq R* package were used to determine the significant enrichment of specific genes in the GO subcategories, and an adjusted Q-value < 0.05 was chosen as the significance cutoff.

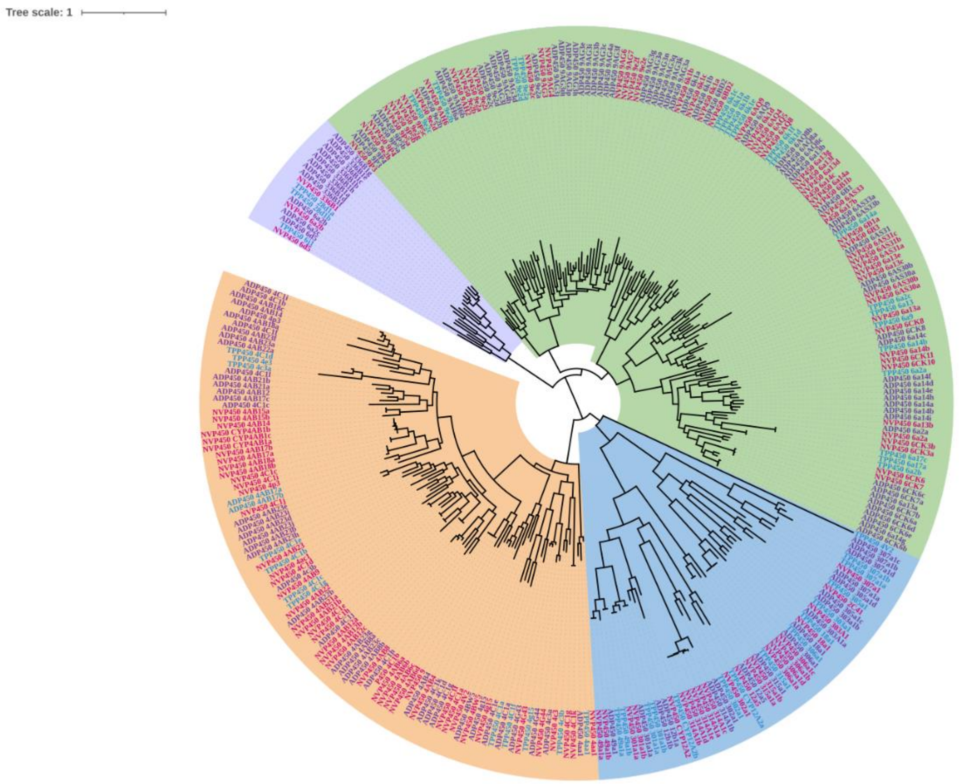

Figure S3. Cytochrome P450 genes in the *Anastatus disparis* genome. The maximum-likelihood phylogenetic tree of P450 proteins is shown for three chalcidoids *A. disparis* (AD), *Nasonia vitripennis* (NV)and *Trichogramma pretiosum* (TP) using the IQ-TREE package.

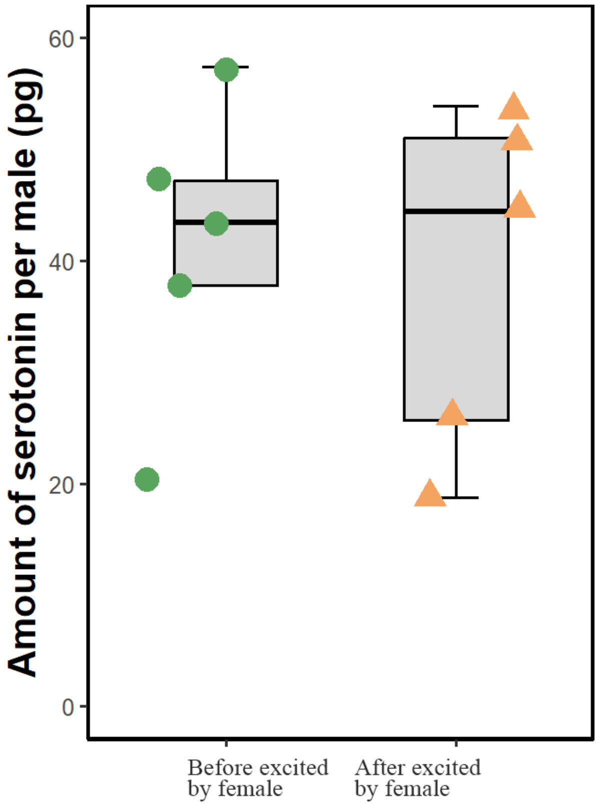

Figure S4. Amount of serotonin in male excited by female. To acquire samples of males excited by female for detecting the amount of serotonin, one-day-old virgin male was introduced into a petri dish containing one-day-old mated female for 30 mins and then collected. Serotonin level in *A. disparis* male brains was measured using an ELISA kit (E-EL-0033, Elabscience, China) following the manufacturer's protocols. Each group contained 25 male heads, which were removed and dissected freshly in PBS. There were 5 biological replicates for each sample.

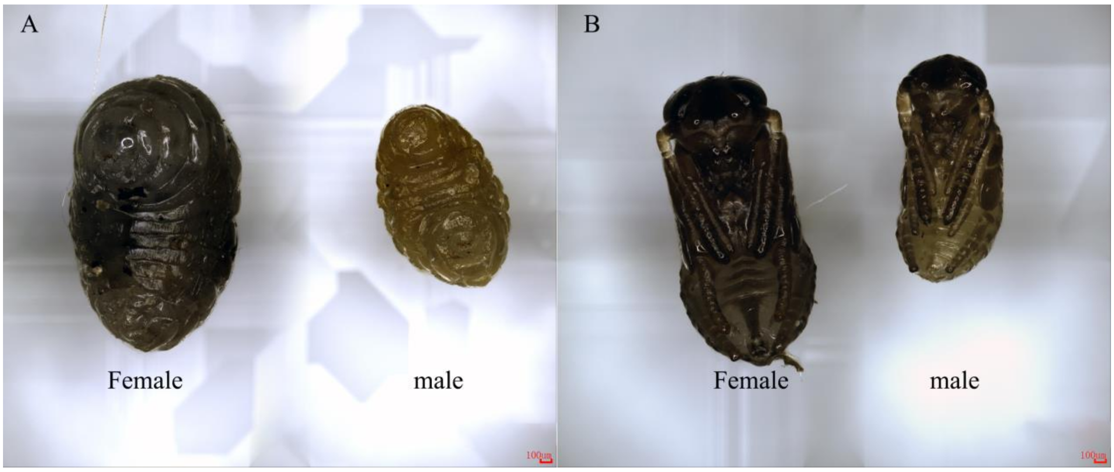

Figure S5. Photograph of *Anastatus disparis* at the pre-pupal and pupal developmental stages.

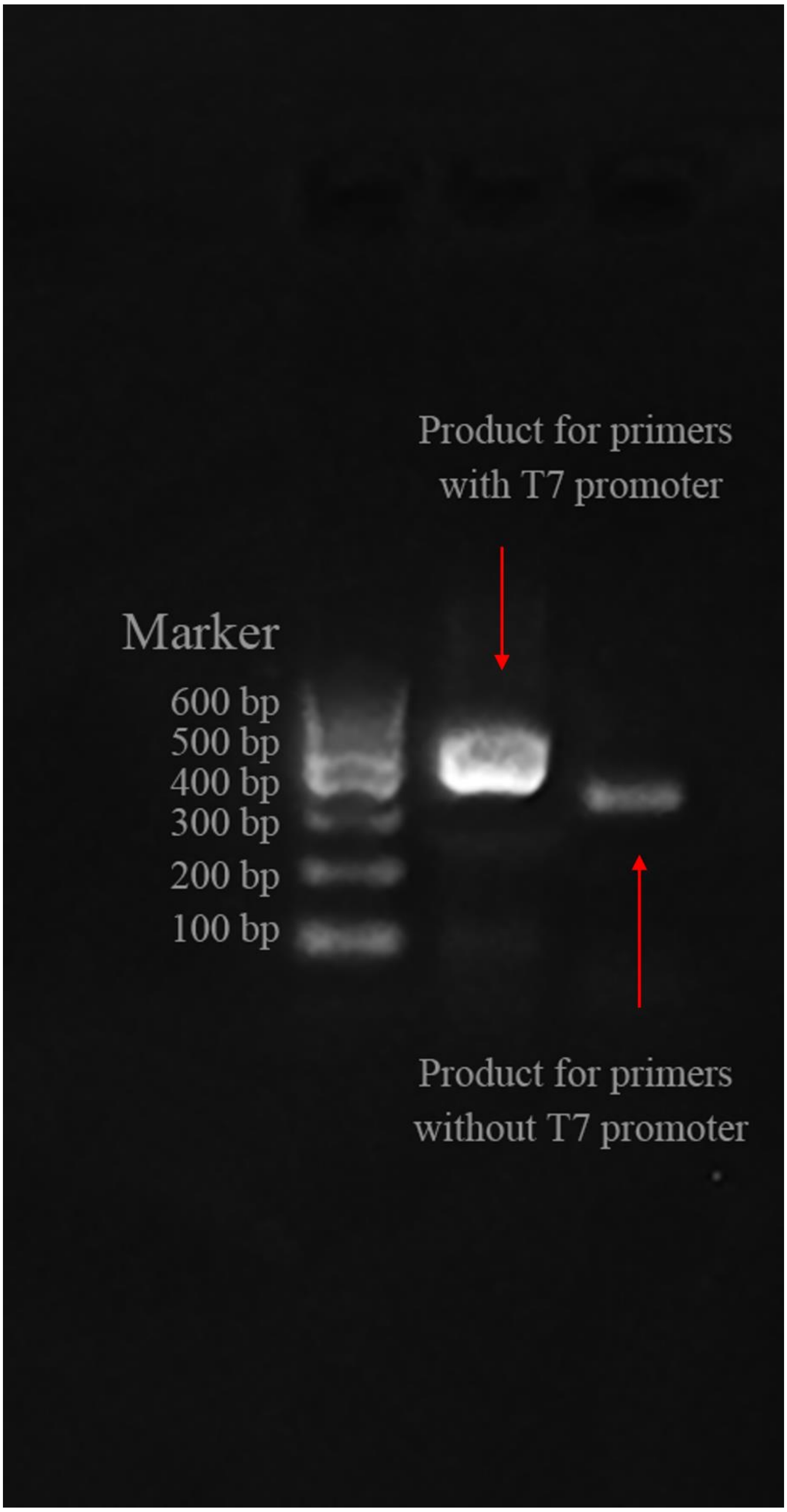

Figure S6. Gel electrophoresis for checking the product of *dsRNAApoLp*. Two PCR products were obtained from the primers with and without the T7 promoter.
