# Supplementary material for: The Chromosome-level Genome Provides Insights into the Evolution and Adaptation of Extreme Aggression
Source: Mol Biol Evol. 2024 Sep 13;41(9):msae195. doi: 10.1093/molbev/msae195 (PMC11427683; doi:10.1093/molbev/msae195)
Supplement: msae195_Supplementary_Data [file msae195_supplementary_data.zip › Supplementary Table 1-Tabale 18.pdf]

**Table S1. Clean data statistics of *Anastatus disparis* genome**

| Data type | Sequence Number | Sum Base (bp) | N50 length (bp) | N90 Length (bp) | Mean Length (bp) | Max Length (bp) | Mean Quality score |
|---|---|---|---|---|---|---|---|
| Clean data | 5,735,295 | 91,357,227,837 | 20,733 | 9,162 | 15,928 | 340,428 | 8.94 |

**Table S2. Primary genome assembly of *Anastatus disparis***

| Contig number (>1Kb) | Contig length (bp) (>1Kb) | Contig N50 (bp) | Contig N90 (bp) | Contig max (bp) | GC content (%) | Gap total length (bp) |
|---|---|---|---|---|---|---|
| 408 | 939581021 | 5041440 | 1273406 | 38756149 | 29.50 | 0 |

**Table S3. Genomic features of selected Hymenopteran insects**

| Genomic Features | *Anastatus disparis* | *Nasonia vitripennis* | *Trichogramma pertiosum* | *Apis mellifera* | *Pteromalus puparum* |
|---|---|---|---|---|---|
| Genome size (Mb) | 939.58 | 295.78 | 195.09 | 225.2 | 338.1 |
| GC content (%) | 29.5 | 41.7 | 39.9 | 32.5 | 40.62 |
| Repeat content (%) | 65.23 | 20.63 | 30.3 | 5.28 | 40.11 |
| Number of protein-coding genes | 19,246 | 24,388 | 12,928 | 9,935 | 14,946 |

**Table S4. Genome assembly at the chromosomal level by Hi-C**

| Item | Value |
|---|---|
| Total length (bp) | 939,626,903 |
| Total length without N (bp) | 939,582,303 |
| Total number of scaffold | 86 |
| GC content (%) | 29.50 |
| N50 (bp) | 183,274,343 |
| N90 (bp) | 170,354,989 |
| Average (bp) | 10,925,894.22 |
| Median (bp) | 25,000.00 |
| Min (bp) | 633 |
| Max (bp) | 220,163,934 |

**Table S5. Length of Chromosome in *Anastatus disparis***

| Chromosome | Length(bp) | contig number |
|---|---|---|
| chr1 | 220,163,934 | 105 |
| chr2 | 190,894,825 | 73 |
| chr3 | 183,274,343 | 128 |
| chr4 | 171,821,451 | 71 |
| chr5 | 170,354,989 | 74 |
| chrUnn | 3,117,361 | 81 |

**Table S6. Quality assessment of genome assembly and annotation using BUSCO**

| | Complete BUSCOs | Complete and single copy BUSCOs | Complete and duplicated BUSCOs | Fragmented BUSCOs | Missing BUSCOs | Total Lineage BUSCOs |
|---|---|---|---|---|---|---|
| Assembly | 1313 (96.05%) | 1291 (94.44%) | 22 (1.61%) | 18 (1.32%) | 36 (2.63%) | 1,367 |
| Annotation | 1315 (96.20%) | 1285(94.00%) | 30 (2.19%) | 20 (1.46%) | 32 (2.34%) | 1367 |

**Table S7. Assessment the gene coverage with the transcriptome data**

| Species | Total reads | Mapped reads | Mapped (%) | Properly mapped reads | Properly mapped (%) |
|---|---|---|---|---|---|
| *Anastatus disparis* | 284,827,588 | 283,068,693 | 99.38 | 276,122,056 | 97.27 |

**Table S8. Repeat sequences in *Anastatus disparis* genome**

| Type | Number | Length | Rate (%) |
|---|---|---|---|
| ClassI | 1,029,103 | 408,104,751 | 43.43 |
| ClassI/DIRS | 10,065 | 4,191,484 | 0.45 |
| ClassI/LARD | 430,340 | 142,307,365 | 15.15 |
| ClassI/LINE | 135,492 | 66,459,843 | 7.07 |
| ClassI/LTR/Copia | 89,035 | 40,183,616 | 4.28 |
| ClassI/LTR/Gypsy | 266,574 | 147,199,206 | 15.67 |
| ClassI/LTR/Unknown | 43,635 | 21,629,272 | 2.30 |
| ClassI/PLE | 35,809 | 15,708,255 | 1.67 |
| ClassI/SINE | 8,265 | 2,165,276 | 0.23 |
| ClassI/TRIM | 7,244 | 6,724,265 | 0.72 |
| ClassI/Unknown | 2,644 | 843,246 | 0.09 |
| ClassII | 372,692 | 170,866,284 | 18.19 |
| ClassII/Crypton | 903 | 184,185 | 0.02 |
| ClassII/Helitron | 105,247 | 49,050,157 | 5.22 |
| ClassII/MITE | 15,382 | 6,224,218 | 0.66 |
| ClassII/Maverick | 48,634 | 48,733,715 | 5.19 |
| ClassII/TIR | 161,291 | 57,352,813 | 6.10 |
| ClassII/Unknown | 41,235 | 14,412,330 | 1.53 |
| Protential Host Gene | 33,114 | 9,666,372 | 1.03 |
| SSR | 31,052 | 18,387,924 | 1.96 |
| Unknown | 231,755 | 82,571,895 | 8.79 |
| Total | 1,697,716 | 612,899,621 | 65.23 |

**Table S9. Protein-coding genes predicted in the genome of *Anastatus disparis***

| Method | Software | Species | Gene number |
|---|---|---|---|
| Ab initio | Genscan | - | 14,454 |
| | Augustus | - | 23,159 |
| | GlimmerHMM | - | 87,660 |
| | GeneID | - | 16,062 |
| | SNAP | - | 115,351 |
| Homology-based | GeMoMa | *Apis mellifera* | 9,884 |
| | | *Athalia rosae* | 10,982 |
| | | *Nasonia vitripennis* | 17,814 |
| | | *Macrocentrus cingulum* | 16,530 |
| RNAseq | TransDecoder | - | 16,670 |
| | GeneMarkS-T | - | 3,071 |
| | PASA | - | 13,520 |
| Integration | EVM | - | 19,246 |

**Table S10. Basic information of *Anastatus disparis* genome**

| Gene Number | Gene length | Average Gene length | Exon Length | Average Exon Length | Exon Number | Average Exon Number | CDS Length | Average CDS length | CDS Number | Average CDS Number |
|---|---|---|---|---|---|---|---|---|---|---|
| 19,246 | 135,723,588 | 7,052.04 | 38,047,394 | 1,976.90 | 104,333 | 5.42 | 27,512,232 | 1,429.50 | 101,147 | 5.26 |

**Table S11. Noncoding RNAs in *Anastatus disparis* genome**

| RNA classification | Number |
|---|---|
| miRNA | 40 |
| rRNA | 114 |
| tRNA | 280 |

**Table S12. Pseudogene in *Anastatus disparis* genome**

| Software | Number | Total length | Average length |
|---|---|---|---|
| GeneWise | 3,589 | 9,408,007 | 2,621.34 |

**Table S13. Functional annotation of *Anastatus disparis* genome**

| Annotation database | Annotated number | Percentage (%) |
|---|---|---|
| GO Annotation | 6,502 | 33.78 |
| KEGG Annotation | 6,467 | 33.60 |
| KOG Annotation | 10,003 | 51.97 |
| TrEMBL Annotation | 17,508 | 90.97 |
| Nr Annotation | 17,383 | 90.32 |
| All Annotated | 17,621 | 91.56 |

**Table S14. Gene family clusters among *Anastatus disparis* and 11 other insect species**

| Item | Acep | Adis | Amel | Aros | Cflo | Csol | Dmel | Fari | Mcin | Nvit | Tcas | Tper |
|---|---|---|---|---|---|---|---|---|---|---|---|---|
| Number of genes | 10,251 | 19,246 | 9,881 | 10,035 | 12,015 | 9,702 | 13,576 | 10,870 | 11,993 | 13,578 | 12,814 | 12718 |
| Number of genes in orthogroups | 9,316 | 15,974 | 8,929 | 8,998 | 10,481 | 8,688 | 9,228 | 9,695 | 9,591 | 11,999 | 10,325 | 11070 |
| Number of unassigned genes | 935 | 3,272 | 952 | 1,037 | 1,534 | 1,014 | 4,348 | 1,175 | 2,402 | 1,579 | 2,489 | 1648 |
| Percentage of genes in orthogroups | 90.9 | 83.0 | 90.4 | 89.7 | 87.2 | 89.5 | 68.0 | 89.2 | 80.0 | 88.4 | 80.6 | 87.0 |
| Percentage of unassigned genes | 9.1 | 17.0 | 9.6 | 10.3 | 12.8 | 10.5 | 32.0 | 10.8 | 20.0 | 11.6 | 19.4 | 13.0 |
| Number of orthogroups containing species | 8,365 | 10,058 | 8,390 | 8,142 | 8,481 | 8,011 | 6,747 | 8,625 | 7,934 | 9,442 | 7,942 | 8416 |
| Percentage of orthogroups containing species | 51.8 | 62.2 | 51.9 | 50.4 | 52.5 | 49.6 | 41.8 | 53.4 | 49.1 | 58.4 | 49.1 | 52.1 |
| Number of species-specific orthogroups | 45 | 755 | 29 | 36 | 162 | 21 | 444 | 100 | 342 | 240 | 320 | 179 |
| Number of genes in species-specific orthogroups | 202 | 2,977 | 130 | 122 | 552 | 63 | 1,546 | 358 | 1,064 | 853 | 1,288 | 1168 |
| Percentage of genes in species-specific orthogroups | 2.0 | 15.5 | 1.3 | 1.2 | 4.6 | 0.6 | 11.4 | 3.3 | 8.9 | 6.3 | 10.1 | 9.2 |

Sign: *Acep = A. cephalotes, Adis = A. disparis, Amel = A. mellifera, Aros =A. rosae, Cflo = C. floridanum, Cso l= C. solmsi, Dmel = D. melanogaster, Fari = F. arisanus, Mcin = M. cingulum, Nvit = N. vitripennis, Tcas= T. castaneum, Tper = T. pretiosum*

**Table S15. GO enrichment analysis of DEGs during male–male aggression**

| Groups | Ontology | GO ID | GO term | p value |
|---|---|---|---|---|
| Aggression for 30 mins | Molecular Function | GO:0016491 | oxidoreductase activity | 0.032 |
| Aggression for 60 mins | Cellular Component | GO:0005576 | extracellular region | 0.019 |
| | Molecular Function | GO:0005319 | lipid transporter activity | 0.033 |

**Table S16. Statistics of transcriptome clean reads and mapped ratio**

| Experiment Number | sex | Sample name | Clean reads | GC Content | Mapped ratio |
|---|---|---|---|---|---|
| No. 1 | Male | Isolation | 21,886,690 | 37.61% | 90.75% |
| | Male | | 19,719,486 | 36.30% | 95.40% |
| | Male | | 19,719,486 | 36.43% | 94.93% |
| | Male | Excited by female for 30 mins | 20,890,320 | 36.25% | 94.55% |
| | Male | | 21,195,472 | 34.82% | 94.54% |
| | Male | | 21,137,732 | 36.00% | 94.63% |
| No. 2 | Male | Isolation | 21,086,911 | 38.46% | 87.67% |
| | Male | | 23,581,116 | 37.14% | 92.38% |
| | Male | | 21,069,226 | 38.93% | 87.88% |
| | Male | Aggression for 30 mins | 20,930,151 | 39.27% | 83.88% |
| | Male | | 20,462,871 | 36.56% | 93.72% |
| | Male | | 23,906,802 | 43.81% | 93.10% |
| | Male | Aggression for 60 mins | 21,886,690 | 37.61% | 90.75% |
| | Male | | 19,719,486 | 36.30% | 95.40% |
| | Male | | 21,330,869 | 36.43% | 94.93% |

## Table S17. Primer pairs used for expression analysis using qRT-PCR

| Gene name | Forward | Reverse |
|---|---|---|
| *Rdx-like-X1* | 5’-CGTTTGGTGGCGTTTAGGAT-3’ | 5’-CTGAACGCTCTGACTGTTGG-3’ |
| *Rdx-X1* | 5’-AGTGGCAATCAAAGCGATGT-3’ | 5’-TTTCTTCCAGCAGGCCATTT-3’ |
| *Rdx-X2* | 5’-AGCGTGGAAACAGCCAATTC-3’ | 5’-AATCAGTTGGTTGAGAAGGA-3’ |
| *Rdx-X3* | 5’-TTTGCGGCTCTGTTTGATTT-3’ | 5’-TCAACAGCTAATGCCTCAATGT-3’ |
| *Rdx-X4* | 5’-TTCGCTTCATGAGGGCAAAT-3’ | 5’-TGGCGTGGCTTCTATTCTTG-3’ |
| *Rdx-like-X2* | 5’-GCATGCGACACTCTTGTTCT-3’ | 5’-TGCAACATCAGAAAGAATTTGTGC-3’ |
| *Rdx-X5* | 5’-AGGTGGCTCACTGATGTGAA-3’ | 5’-TTGGCGTAGAGAGCTTGTGA-3’ |
| *Rdx-X6* | 5’-CATGTTGCAGTGGTAGCAGA-3’ | 5’-TGCAACATATTGTCAGTTGATCGT-3’ |
| *Rdx-X7* | 5’-TGAACTCGTCATTCGTCACA-3’ | 5’-TTCCGTGCTGGAATCCATTT-3’ |
| *Rdx-like-X3* | 5’-AAAGTTAATGCCCACAAAGC-3’ | 5’-AGTGACGAGACTAGACCTGTA-3’ |
| *Rdx-like-X4* | 5’-CAAAGGAACTGCCGATCGAA-3’ | 5’-TTGCCCACCACAAACACAAT-3’ |
| *Rdx-like-X5* | 5’-TGGCGGATATGTAATGCTTTCG-3’ | 5’-CACGTCAGAATCCGATTGGT-3’ |
| *Rdx-like-X6* | 5’-TAGAGGAAATGGACGCTGCT-3’ | 5’-AAGATCATGGACGCCGTATT-3’ |
| *Rdx-X8* | 5’-AGGAACCATCTTTCAGTCTCCA-3’ | 5’-AAACTGCACACTTCTTTCGT-3’ |
| *Rdx-X9* | 5’-GCGAACAAACGTTAGCATGT-3’ | 5’-TGGTCTTAACCACTTGTTCCCT-3’ |
| *Rdx-like-X7* | 5’-GGATTGTCCGCTGACATGAG-3’ | 5’-CGCTAGCGTCCATTTCCTTT-3’ |
| *Rdx-X10* | 5’-TGGTTCTTATACTTGGACAATCTACA-3’ | 5’-AGCTTGAACCCAACGGGTAT-3’ |
| *Rdx-X11* | 5’-TCAGTGGAAACGAACGAACA-3’ | 5’-ACATTCATTGTCCAAACAAGC-3’ |
| *Rdx-like-X8* | 5’-AAGACCACTGATGGTGGGAA-3’ | 5’-CCTGCTTATAAACTTCCTGAATCCC-3’ |
| *Rdx-like-X9* | 5’-ATTGAGCATTTGCACAGACG-3’ | 5’-TCTTTGAGCACCACGGGTTT-3’ |
| *Rdx-like-X10* | 5’-TCACTTGAAAGTTGTTGATCCT-3’ | 5’-CCCTTCAAGTGGCTTTCGTT-3’ |
| *Rdx-X12* | 5’-TGTGGAAGAGAACAACCTGA-3’ | 5’-AAGGCTTTGCCAACTCTTCA-3’ |
| *TH* | 5’-AGTCTGCTCTTGGCCATCAT-3’ | 5’-GACGATGCACGCTTTGAAAC-3’ |
| *Tret1-like* | 5’-TTGCTGTGGCTGCTTCATTT-3’ | 5’-ACTGCGTAAACGTGAAGGTG-3’ |
| *AKR1A1b-like* | 5’-GGCGATGAAGATACAACGGT-3’ | 5’-TCTGGTCTGTTGCCGTATGA-3’ |
| *iPLA(2)* | 5’-CGAGACCGCTTACAGAGAGT-3’ | 5’-GCTGACCAAGCAGCTTCAAT-3’ |
| *agl-like* | 5’-TAGACCACCTGGACCACCTA-3’ | 5’-TGCCATCACCATCGCTATCT-3’ |
| *Vg3-like* | 5’-ACAAGAGGACGAGGTTGTGT-3’ | 5’-TTCACCTTGGTGTAGACGCT-3’ |
| *PLA2* | 5’-CGGACCTCATAGGTTAGCGT-3’ | 5’-CAATGGCTAAGGGTGAACGG-3’ |
| *FAS-like* | 5’-CGCTTATGCAGATGGCTGTT-3’ | 5’-CCAACTGCTGCCATAGAACC-3’ |
| *4CL-like* | 5’-ACCACATCTCGCCTTCTCAA-3’ | 5’-CCGGGAACCTTGACTACGAA-3’ |
| *SCAD* | 5’-TGTGCAAGTTGTGGCGTTAT-3’ | 5’-GCTTTAGCCATTGTTGATGCAG-3’ |
| *stp-like* | 5’-GAGCTTGCACAGATGCAGAA-3’ | 5’-TGGAAGACCATTCCAGCACA-3’ |
| *Lip 1* | 5’-TACTTATCGGGCCTGGCAAA-3’ | 5’-TACATCGCGTGTTGCCATTT-3’ |
| *ApoLp* | 5’-GGCAAACGTCTTGGTGAACT-3’ | 5’-TGATTTGAGGTCGGCTCCTT-3’ |
| *GP* | 5’-CGCTTGGTCTTGCTGCTTAT-3’ | 5’-ATTCAGGTCTGGCCTTCTCC-3’ |
| *ADΔ11* | 5’-TGGGTAAAGATTCATCGGACAC-3’ | 5’-AAGAAGCGCCAACCAATGTG-3’ |
| *GCDH-like* | 5’-CCCAGCAAGCGATCCTAATG-3’ | 5’-GCACCTTTCTCGCCAATCAT-3’ |
| *LPIN1* | 5’-ATTAATGCCGCAACCCTCAC-3’ | 5’-CCAAAGCGTACATGGAAGGG-3’ |
| *LRP4* | 5’-ACTGCACTGCGGAACAATTT-3’ | 5’-TGCACATCCGCTAAGCTTTC-3’ |
| *AKR1A1a-like* | 5’-CAGCAGAGAAAGCAGTGACA-3’ | 5’-ACATCTGAAGGTCGCATAGC-3’ |
| *ACBP4* | 5’-ATGTATAATATTTTTGCTT-3’ | 5’-TTAAGTTTCTTTAAATTCC-3’ |

| | | |
|---|---|---|
| *POA3-like* | 5’-GGCCTCGTACTTCTCCCTTT-3’ | 5’-GAAGGAAGGCAGCATAAGCC-3’ |
| *EF1A* | 5’-ACCACGAAGCTCTCCAAGAA-3’ | 5’-AATCTGCAGCACCCTTAGGT-3’ |

**Table S18. Primer sequences for dsRNA synthesis**

| Gene name | Primer |
|---|---|
| *apoLp* | Forward : 5’- ATGGCACTACCACCCCGGCT-3’ |
| | Reverse: 5’- GTCTTGGCACCGGCATCACCA-3’ |
