# Supplementary material for: The Chromosome-level Genome Provides Insights into the Evolution and Adaptation of Extreme Aggression
Source: Mol Biol Evol. 2024 Sep 13;41(9):msae195. doi: 10.1093/molbev/msae195 (PMC11427683; doi:10.1093/molbev/msae195)
Supplement: msae195_Supplementary_Data [file msae195_supplementary_data.zip › Supplementary Video Notes.pdf]

Supplementary Video Notes

Supplementary Video 1. Video of aggressive chasing behavior resulting in one male injury. Injured males partially lose their midleg and exhibit mobility difficulties and struggles.

Supplementary Video 2. Video of aggressive behavior upon sneak attack by an opponent.

Supplementary Video 3. Video of aggressive behavior on the boxing.
